# Wearable Wireless Power Systems for “ME-BIT” Magnetoelectric-Powered Bio Implants

**DOI:** 10.1088/1741-2552/ac1178

**Published:** 2021-07-26

**Authors:** Fatima T. Alrashdan, Joshua C. Chen, Amanda Singer, Benjamin W. Avants, Kaiyuan Yang, Jacob T. Robinson

**Affiliations:** Rice University, Houston, TX 77005, USA.; Rice University, Houston, TX 77005, USA.; Rice University, Houston, TX 77005, USA.; Rice University, Houston, TX 77005, USA.; Rice University, Houston, TX 77005, USA.; Rice University, Houston, TX 77005, USA.; Baylor College of Medicine, Houston, TX 77030, USA.

**Keywords:** Wearable system, implantable device, bioelectronics, magnetoelectric effect, wireless power transfer

## Abstract

**Objective.:**

Compared to biomedical devices with implanted batteries, wirelessly powered technologies can be longer-lasting, less invasive, safer, and can be miniaturized to access difficult-to-reach areas of the body. Magnetic fields are an attractive wireless power transfer (WPT) modality for such bioelectronic applications because they suffer negligible absorption and reflection in biological tissues. However, current solutions using magnetic fields for mm-sized implants either operate at high frequencies (*>*500 kHz) or require high magnetic field strengths (*>*10 mT), which restricts the amount of power that can be transferred safely through tissue and limits the development of wearable power transmitter systems. Magnetoelectric (ME) materials have recently been shown to provide a wireless power solution for mm-sized neural stimulators. These ME transducers convert low magnitude (*<*1 mT) and low-frequency (~300 kHz) magnetic fields into electric fields that can power custom integrated circuits or stimulate nearby tissue.

**Approach.:**

Here we demonstrate a battery-powered wearable magnetic field generator that can power a miniaturized MagnetoElectric-powered Bio ImplanT “ME-BIT” that functions as a neural stimulator. The wearable transmitter weighs less than 0.5 lbs and has an approximate battery life of 37 hr.

**Main results.:**

We demonstrate the ability to power a millimeter-sized prototype “ME-BIT” at a distance of 4 cm with enough energy to electrically stimulate a rat sciatic nerve. We also find that the system performs well under translational misalignment and identify safe operating ranges according to the specific absorption rate limits set by the IEEE Std 95.1–2019.

**Significance.:**

These results validate the feasibility of a wearable system that can power miniaturized magnetoelectric implants that can be used for different neuromodulation applications.

## I. Introduction

Bioelectronics is transforming the medical field through engineered implants that can deliver precise, personalized, and long term therapies that are difficult to achieve using traditional pharmaceuticals [1]–[7]. One example application for bioelectronics is neural stimulation. Neural stimulators modulate the electrical activity of the nervous system providing effective treatment for a wide range of neurological and psychiatric disorders [8]–[12].

Miniaturization is an important goal for bioelectronic devices like neural stimulators. By making devices smaller, one can reduce invasiveness and improve accessibility to difficult-to-reach areas of the body. However, on-board batteries used to power a device are often a bottleneck in reducing the device footprint. Moreover, the infections, movement, and breakage associated with wires that connect the battery and stimulator impose serious risks to the patient’s health and require periodic battery replacement surgeries [13]. To make extremely miniature bioelectronics, it is necessary to eliminate batteries in favor of wireless power and data transfer.

Several methods are proposed in the literature including acoustic [14], electromagnetic [15], [16], and magnetic technologies with the latter comprising magnetothermal [17], [18], magnetoelectric(ME) [19]–[21], and inductive coupling [22]. While there are advantages to each approach, the physics of wireless data and power transfer imposes performance trade-offs [23]. Using ultrasound waves to transmit both power and data has been demonstrated for miniaturized ‘Stimdust’ motes [14]; however, ultrasound impedance mismatches between bone and tissue typically limit ultrasonic technology to soft tissue applications. In addition, because the ultrasound waves are typically focused by the transmitter, power and data transfer efficiency is sensitive to translational misalignment with the external transceiver. Likewise, the misalignment between the transmitter and the receiver coils limits the power received by the inductively coupled sub-millimeter neurostimulator reported in [22]. While misalignment has minor effects on the wireless power link of the RF implantable rectenna proposed in [16], a high operating frequency limits the amount of power that can be delivered safely to the implant. Although the magnetothermal techniques used to heat magnetic nanoparticles operate in the frequency range of a few hundred kHz where tissue absorption is low, the requirement for a relatively large magnetic field typically more than 10 mT at the location of the nanoparticles requires benchtop field generators that are not easily moved. The large size is due to the fact that these generators typically operate at kW power levels, and are often water-cooled [17], [18].

Magnetoelectrics is a promising solution for millimeter-sized bioelectronic devices due to the large power density and ability to operate with low amplitude magnetic fields (*<*10 mT) at frequencies in the 100–500 kHz band where tissue absorption is low [24]. This technology utilizes a bi-layer thin film of mechanically coupled magnetostrictive and piezoelectric materials that transduce the magnetic field energy to electric energy that can be used to power implanted electronics or stimulate nearby tissues. For example, a mm-sized ME film activated using a low amplitude and low-frequency magnetic field has been used to successfully provide therapeutic deep brain stimulation in a freely moving rodent model for Parkinson’s disease [19]. Exploiting the CMOS technology, the first programmable neural implant leveraging the ME effect (MagNI) has been proposed in [20], [21], [25]. MagNI has a miniaturized volume of 8.2mm^3^ and can deliver programmable bi-phasic current stimulation for different neurostimulation applications. ME micro-resonators operating at GHz frequencies can also be used to power ultra-small low-power devices [26], [27].

While proof-of-principle ME technologies show promise for wireless bioelectronics, many applications require a portable system that includes a wearable solution for data and power transfer. For instance, the spinal cord stimulator used for chemotherapy-induced pain could need up to 6–12 hours of use per day [28]. Likewise, patients must use an auricular vagus nerve stimulator used for depression and epilepsy treatment [29] and chronic pain [30] daily for at least 3–4 sessions.

To design a wearable system for ME implants, it’s important to understand the working principles of both the transmitter and receiver and the design challenges associated with the coupling between them. Also, one should take into account the misalignment issues that are inevitable due to relative body motion, the movement of the device inside the body, and the difficulty that comes with patients wearing the transmitter belt to align the system properly. Moreover, since ME technology utilizes magnetic fields for power transfer, it’s important to ensure that the field exposure has no adverse effects on the user’s health.

In our previous work [20], [21], we have proposed a conceptual design for a transmitter system that can be assembled in a wearable belt and power ME implants for spinal cord stimulation applications. In this paper, we demonstrate a prototype of a battery-powered wearable system capable of generating a low-amplitude (~1 mT) and low-frequency (~100s kHz) magnetic field sufficient to generate several mW of power in millimeter-sized magnetoelectric implant at a distance up to 4 cm. We first present the receiver and transmitter design theory and compare the simulation results to the experimental results. We then show the experimental results of the overall system performance at different distances, and translational misalignments. To validate the efficacy of our system in an in vivo environment, we demonstrate its ability to electrically stimulate the sciatic nerve of a rat with a transmitter-to-receiver distance of 4 cm. We further verify the system’s compliance with the IEEE C95.1–2019 safety standard by using COMSOL multiphysics software to model the brain and study the effects of magnetic field exposure on the implant’s surrounding tissue.

## II. System overview

**Fig. 1.**
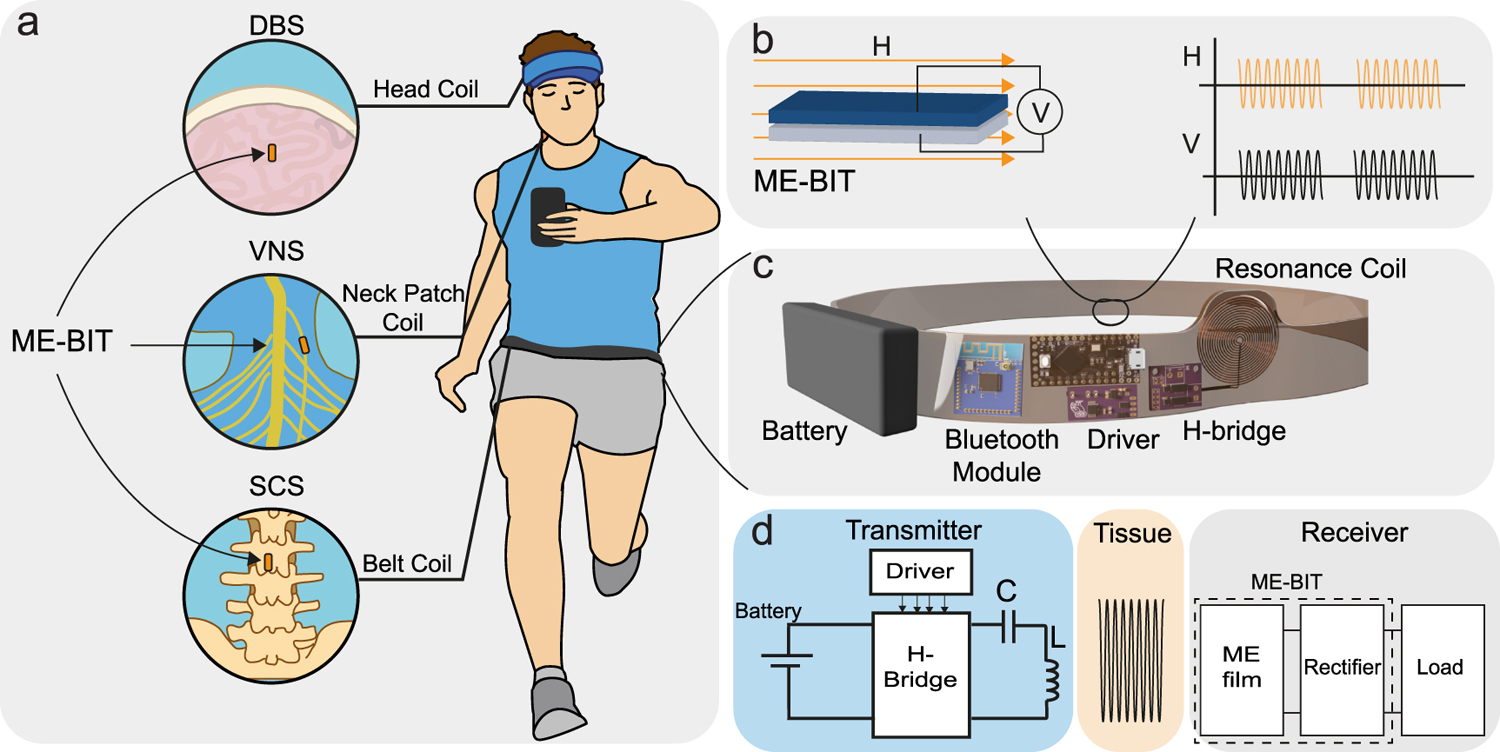
Conceptual illustration of the wearable system, (a)applications including DBS,VNS, and SCS by adjusting the transmitter coil position in the head, neck, and back respectively, (b)ME receiver working principle, (c)prototype of the wearable transmitter system, (d)block diagram of the overall system.

Here we describe a proof-of-principle wearable wireless power transfer (WPT) system for **M**agneto-**E**lectric-powered **B**io **I**mplan**T** or “ME-BIT” (Fig. 1). We define these general ME-powered implants as having a ME material to harvest data and power, and a rectifier circuit (Fig. 1.b,d). To power these millimeter-sized bioelectronic implants we designed a light-weight transmitter that combines custom drive electronics, wire coil, batteries, and a Bluetooth module integrated into a wearable belt as shown in Fig. 1.c. This transmitter system weighs less than 0.5 lbs and can generate a sufficient magnetic field to power the ME-BIT at an implantation depth of up to 4 cm.

In addition to providing power to the implant, this system can modulate the amplitude and frequency of the field to send data to the digitally programmable ME-BIT for neural interfaces “MagNI” [20], [21] or to control the stimulation waveform of analog ME-BIT neural simulators [19]. Moreover, the same system can be used to power the ME-BITs used in different neurostimulation applications including the deep brain (DBS), vagus nerve (VNS), and spinal cord stimulation (SCS) by adjusting the transmitter coil position in the head, neck, and back respectively (Fig. 1.a). We will discuss the receiver and transmitter characteristics and design specifications in the following sub-sections.

### A. Receiver

**Fig. 2.**
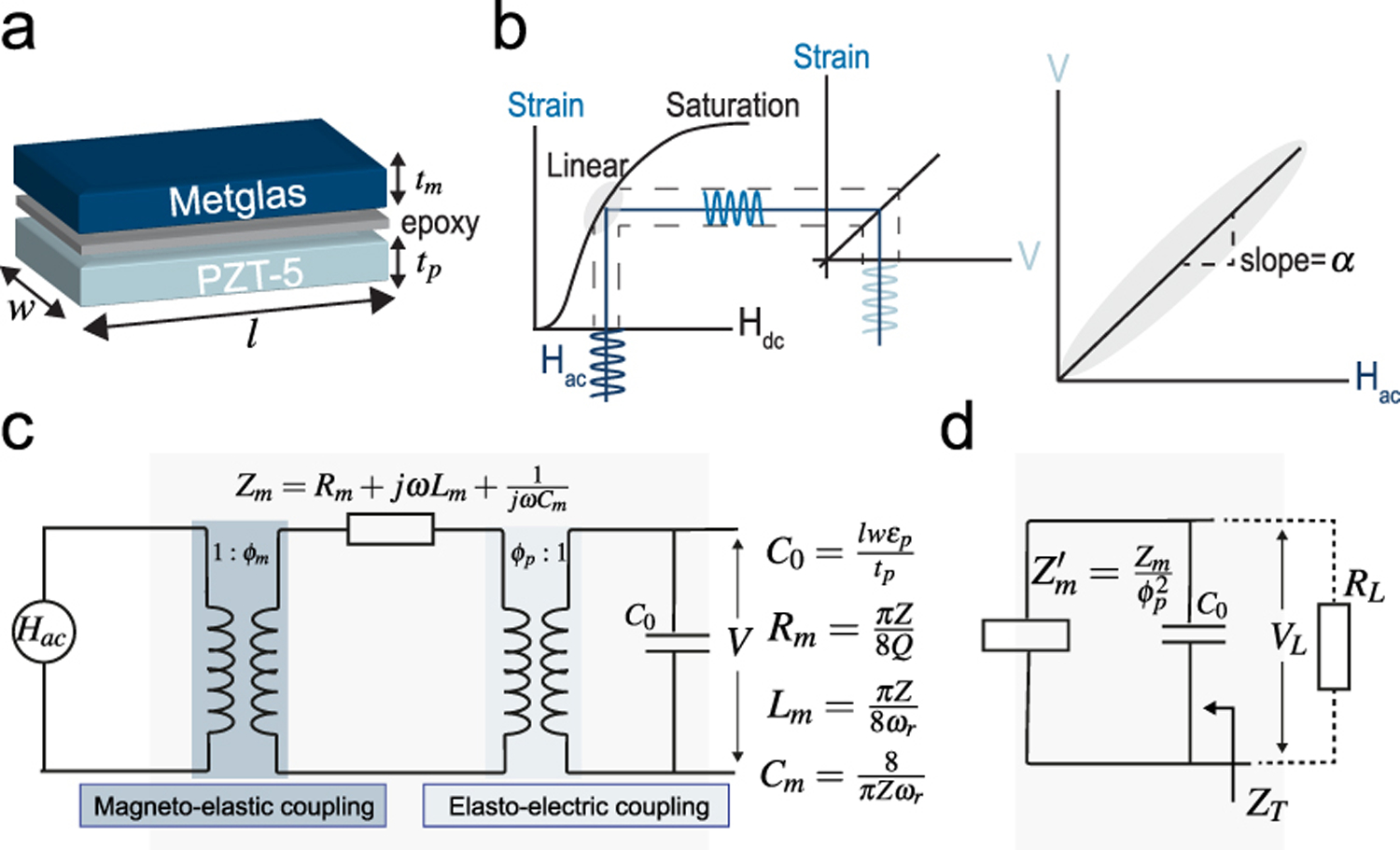
(a)ME film structure, (b)working principle of ME effect, (c) Equivalent circuit model of the ME film, (d) Equivalent impedance of the ME film.

Our ME-BIT utilizes a thin bilayer film of two smart materials, Metglas (magnetostrictive) and PZT-5 (piezoelectric), with the two layers bonded using epoxy as shown in Fig. 2.a. The film length, width, Metglas layer thickness, and PZT-5 layer thickness are denoted by *l, w, t*_*m*_, and *t*_*p*_ respectively. The working principle depends on the material behavior of changing its properties in response to external stimuli like a magnetic field, electric field, or mechanical stress.

Applying an alternating magnetic field *H*_*ac*_ along the longitudinal direction of the Metglas layer excites a longitudinal mechanical strain. Due to the nonlinear characteristics of the magnetostrictive material, a significant mechanical strain can only develop when an additional bias field *H*_*dc*_is applied [31]. An optimal *H*_*dc*_ would shift the operating point of the Metglas layer to the inflection point of the magnetic field-strain curve at which the derivative of mechanical strain with respect to the magnetic field is maximum as shown in Fig. 2.b [32]. Mechanical coupling between the layers leads to the transfer of strain from the Metglas layer to the PZT-5 layer through the interfacial epoxy layer. This coupling induces an alternating electric voltage across the laminate’s thickness. The transduction of the magnetic energy to electric energy through the elastic coupling between the two laminates is what we refer to as the magnetoelectric effect.

For wireless power transfer applications, it is important to quantify the electric energy that can be harvested by the receiver in response to a certain magnetic field as well as evaluating the transduction efficiency and the parameters affecting it. The electric energy harvested by the ME transducer depends on the ME coupling between the two laminates which is usually evaluated using the ME voltage coefficient *α*. *α* is defined as the change in the film’s output electric voltage V in response to a change in the applied magnetic field such that:

(1) $$\alpha =\frac{dV}{dH_{ac}},$$

According to the equivalent circuit model developed in [33], [34] and depicted in Fig. 2.c, the open circuit ME voltage coefficient is expressed as:

(2) $$\alpha =\left|\frac{\phi_{p}}{j\omega C_{0}}\frac{\phi_{m}}{Z_{m}}\right|,$$

Where $\phi_{m}=wt_{m}\frac{d_{m,33}}{s_{m}}$, and $\phi_{p}=w\frac{d_{p,31}}{s_{p}}$ are the magneto-elastic and electro-elastic coupling factors, *C*_0_ is the clamping capacitance of the PZT-5 laminate, and *Z*_*m*_ represents the mechanical impedance.

The mechanical impedance is mainly a function of the mechanical quality factor Q, the resonant frequency $\omega_{r}=\frac{\pi v}{l}$ and *Z* = *Avρ* given that the cross-sectional area *A* = *A*_1_ + *A*_2_ where *A*_1_, *A*_2_ represents the cross-sectional area of the PZT-5 and Metglas laminates respectively, the geometric ratio $n=\frac{A_{1}}{A_{1}+A_{2}}=\frac{t_{p}}{t_{p}+t_{m}}$, the average density $\bar{\rho }=\frac{\rho_{p}+\left(\rho_{m}A_{2}/k\right)}{A_{1}+A_{2}}$ and the sound velocity $v^{2}=\frac{1}{\rho }\left(\frac{n}{s_{p}}+\frac{1-n}{ks_{m}}\right)$. All material constants used to calculate the ME coupling coefficient are defined and listed in Table. I.

#### 1) Factors affecting ME voltage coefficient:

We study the ME-transducer geometric and fabrication properties that affect the ME coupling hence the transduction efficiency of the ME receiver. Specifically we find that the ME voltage coefficient is independent of the area of the film and increases as the interface coupling factor *k*, the thickness ratio *a*, or the mechanical quality factor Q increases. Moreover, the resonance frequency of the film is shifted to the right as the length, interface coupling factor, or thickness ratio decreased whereas it’s independent of the mechanical quality factor.

Our modeling shows that the ME voltage coefficient is a function of the frequency, material properties, and geometry. The ME coupling is also affected by the interfacial epoxy layer characteristics. As reported in [35], the ME coupling coefficient decreases as the thickness of the bonding layer increases, or Young’s modulus of the bonding material decreases. To account for this imperfect coupling, authors in [34] introduced an interface coupling factor *k*, 0 *< k <* 1, defined as the percentage of the mechanical stress transferred from the magnetostrictive layer to the piezoelectric layer.

**Fig. 3.**
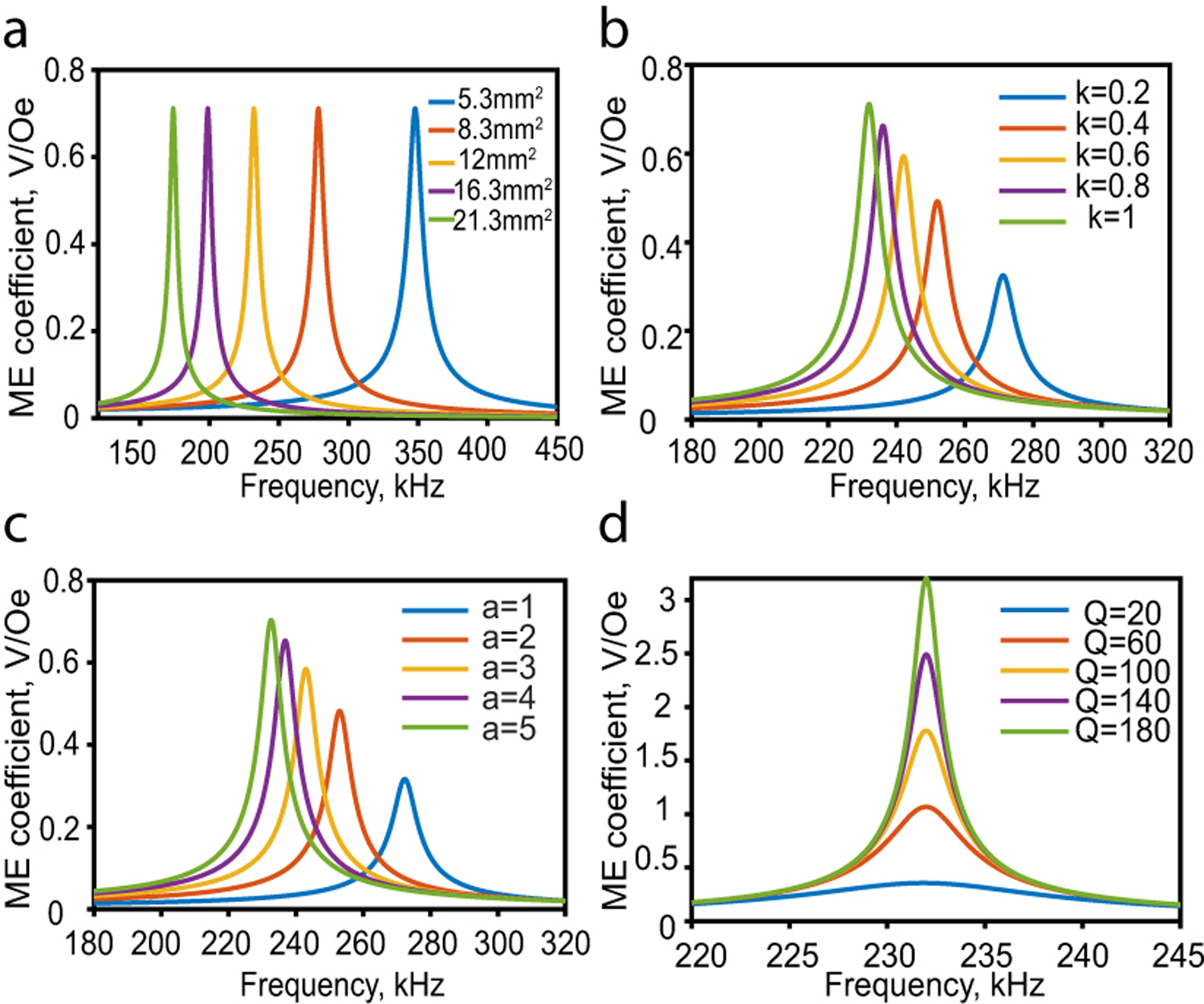
Calculated ME voltage coefficient with a variation of (a)film area where aspect ratio *l*/*w* is fixed to 3, (b)interface coupling factor, (c)thickness ratio, (d)quality factor.

Fig. 3 depicts the general trends of the calculated ME voltage coefficient considering the variation of the area, quality factor, interface coupling factor, and thickness ratio *a* = *t*_*p*_/*t*_*m*_. To begin with, the film is assumed to have a 6mm×3mm perfectly coupled Metglas and PZT-5 layers with a thickness ratio of 5.2, and a quality factor of 35.

The ME voltage coefficient is:

##### a) independent of area:

In Fig. 3.a, the aspect ratio of the film *l*/*w* is fixed to 3, and the length is varied from 4mm to 8mm. While the ME voltage coefficient is not affected by changes in the film area, the resonant frequency decreases as the length increases. Although both length and width are changed, the resonant frequency of the fundamental mode is only determined by the length as defined earlier $\left(\omega_{r}=\frac{\pi v}{l}\right)$.

##### b) increased with larger interface coupling factors :

The bonding layer characteristics significantly affect the film performance as shown in Fig. 3.b, where a stronger coupling between the magnetostrictive and piezoelectric layers results in a higher ME voltage coefficient. However, achieving perfect coupling is limited by the available bonding materials and the fabrication process.

##### c) increased with larger piezoelectric to magnetostrictive thickness ratios:

The ME voltage coefficient is also significantly affected by the ratio between the two laminate thicknesses as shown in Fig. 3.c. A thicker piezoelectric layer compared to the magnetostrictive layer is more desirable.

##### d) increased with larger mechanical quality factors:

A larger mechanical quality factor results in stronger ME coupling as depicted in Fig. 3.d. This factor is usually used to quantify the strain amplification in the ME laminate and is defined as the ratio between the resonant frequency and the 3 dB frequency bandwidth [36]. As can be seen for films with the same resonant frequency, the ones with higher Q have narrower bandwidth.

#### 2) Factors affecting the ME receiver impedance:

In addition to the voltage profile of the ME film, it’s important to study the impedance characteristics to optimize the output power and better match the film with the load impedance. Here we find that, at the resonance frequency, the film impedance

is dominated by the resistive part and it’s magnitude mainly depends on the material properties and film’s geometry.

The equivalent impedance of the film *Z*_*T*_ can be defined as the parallel combination of the mechanical impedance *Z*_*m*_ reflected at the electric part and the clamping capacitor *C*_0_ impedance as shown in Fig. 2.d.:

(3) $$Z_{T}={Z^{\prime }}_{m}//\frac{1}{j\omega C_{0}}$$

**Fig. 4.**
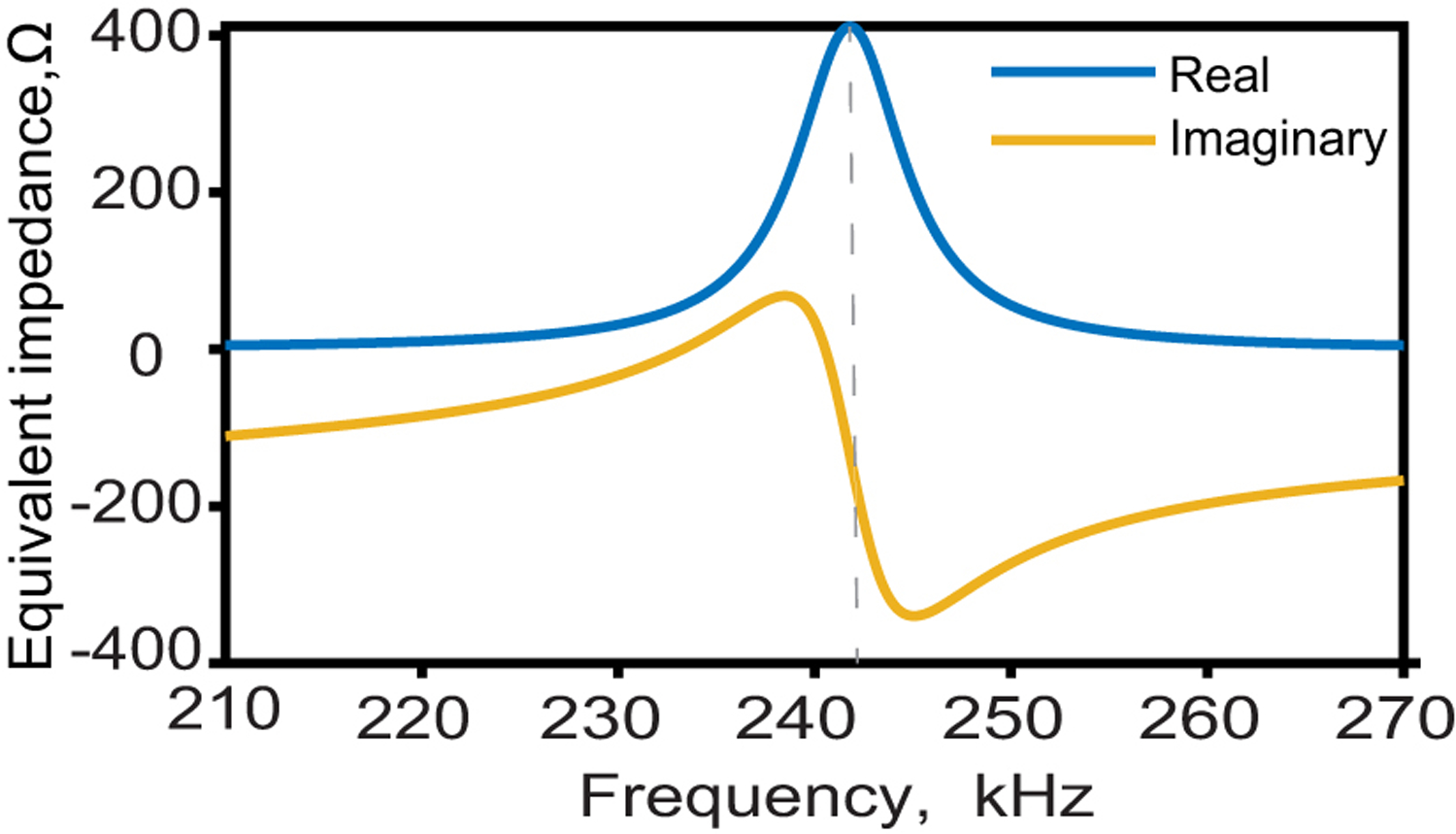
Calculated real and imaginary parts of a 6*3 mm ME film impedance.

Fig. 4 shows the calculated real and imaginary parts of the equivalent impedance of the film defined as the base case previously which has a resonant frequency of 242 kHz. As can be seen, the film impedance is dominated by the real component of the impedance at the resonant frequency, while the imaginary part is more significant off-resonance. Since our system will operate at the resonant frequency to ensure efficient performance, we focused on the characteristics of the real part of the equivalent impedance.

Our model shows that the real impedance is also a function of the frequency, material properties, and geometry. The real impedance magnitude:

##### a) decreases with width and increases with aspect ratio:

**Fig. 5.**
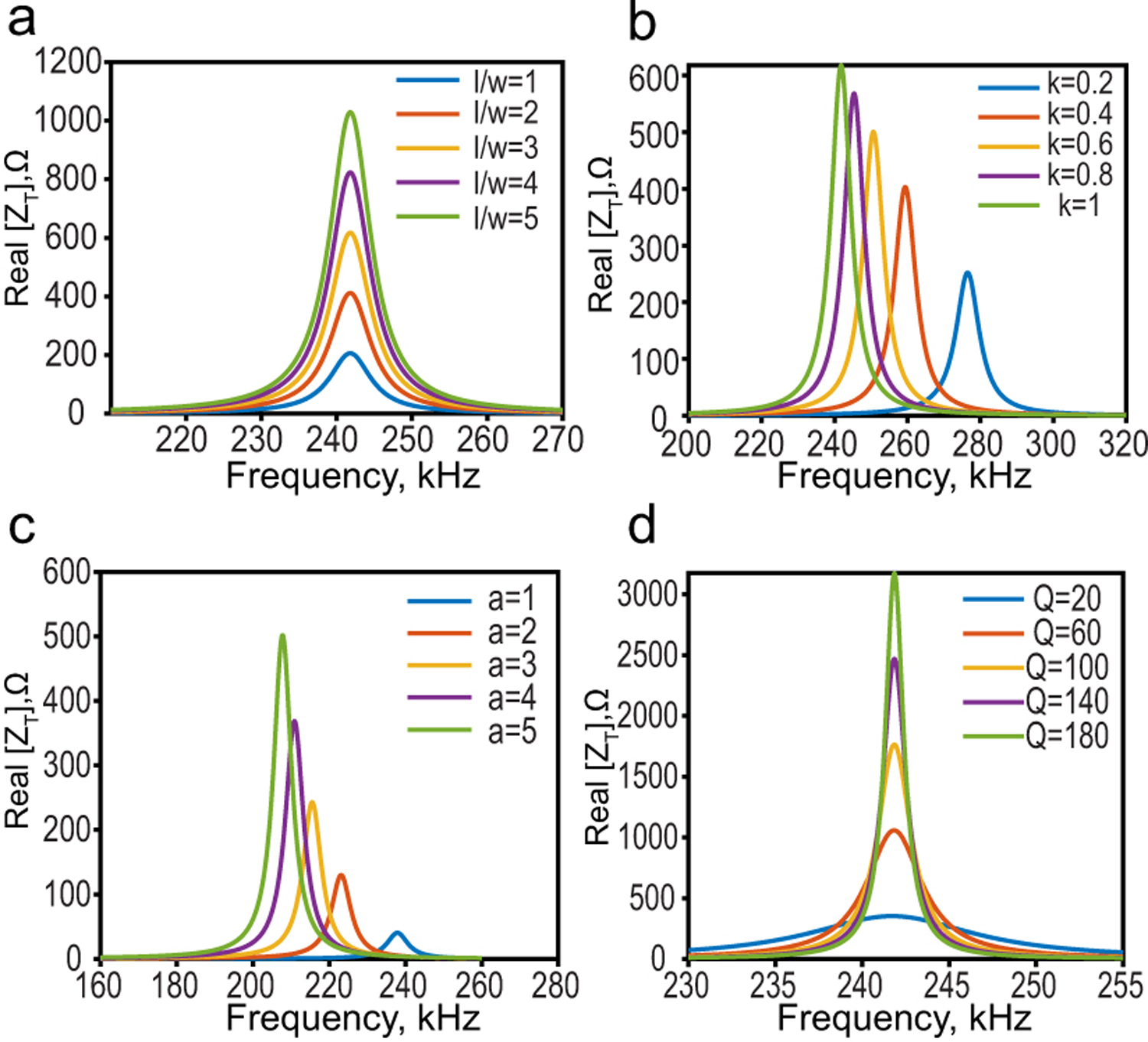
Calculated real impedance with variation of (a)aspect ratio, (b)interface coupling factor, (c)thickness ratio, (d)quality factor.

In Fig. 5.a the length of the film is fixed to 6 mm whereas the width is changed such that the aspect ratio varies from 1 to 5. Clearly, the resonant frequency does not significantly change since the length is fixed (Fig. 5.a), however, the magnitude of the real impedance decreases as the width increases. Also, the impedance is linearly proportional to the aspect ratio.

##### b) increases with larger interface coupling factors :

The interface coupling factor affects both the impedance amplitude and the resonance frequency as shown in Fig. 5.b, a stronger coupling results in larger impedance and a lower resonance frequency.

##### c) increases with with larger piezoelectric to magnetostrictive thickness ratios:

As shown in Fig. 5.c, the thickness ratio can noticeably change the impedance and frequency such that a thicker PZT layer with respect to the Metglas layer results in a larger impedance magnitude and lower resonance frequency.

##### d) increases with larger mechanical Q factor:

as can be seen in Fig. 5.d, the magnitude of the real impedance is linearly proportional to the magnitude of the mechanical quality factor, whereas the resonance frequency is independent of Q.

### B. Transmitter

**Fig. 6.**
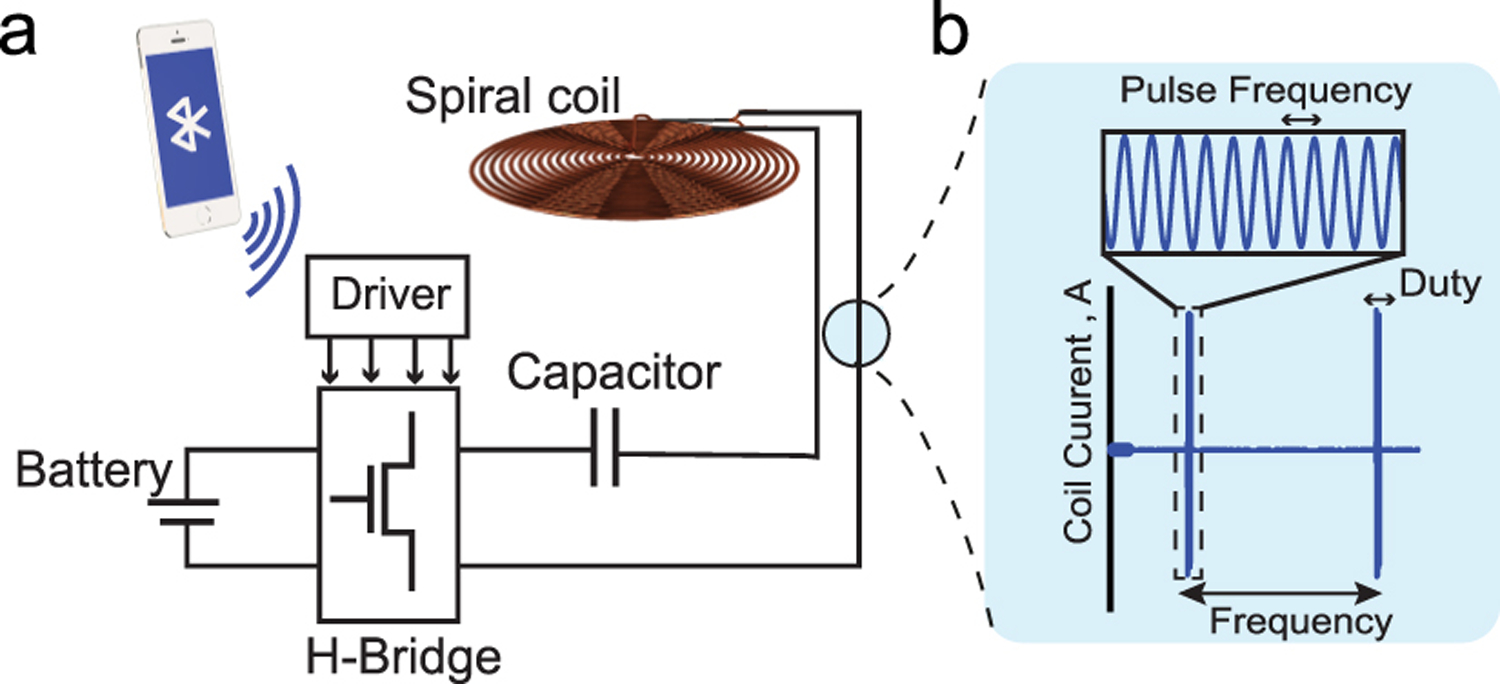
(a)Transmitter system, (b) Coil’s pulsed current parameters.

To generate sufficient magnetic fields to power the implanted ME-BIT, we designed a battery-powered transmitter system as shown in Fig. 6.a. A rechargeable lithium-ion battery connected to an H-bridge converter drives a pulsed alternating current through a resonant circuit of an inductive coil and capacitor bank in series. Three parameters characterize the coil’s pulsed current: the frequency, duty, and pulse frequency as shown in Fig. 6.b. These parameters determine the magnetic field generated by the coil which directly affects the voltage induced at the ME transducer terminal. The user can set these parameters using a mobile application that communicates them through Bluetooth to the driver circuit that combines an HM-19 Bluetooth module, Teensy LC microcontroller, and custom electronics to generate the H-bridge switching signals accordingly.

**Fig. 7.**
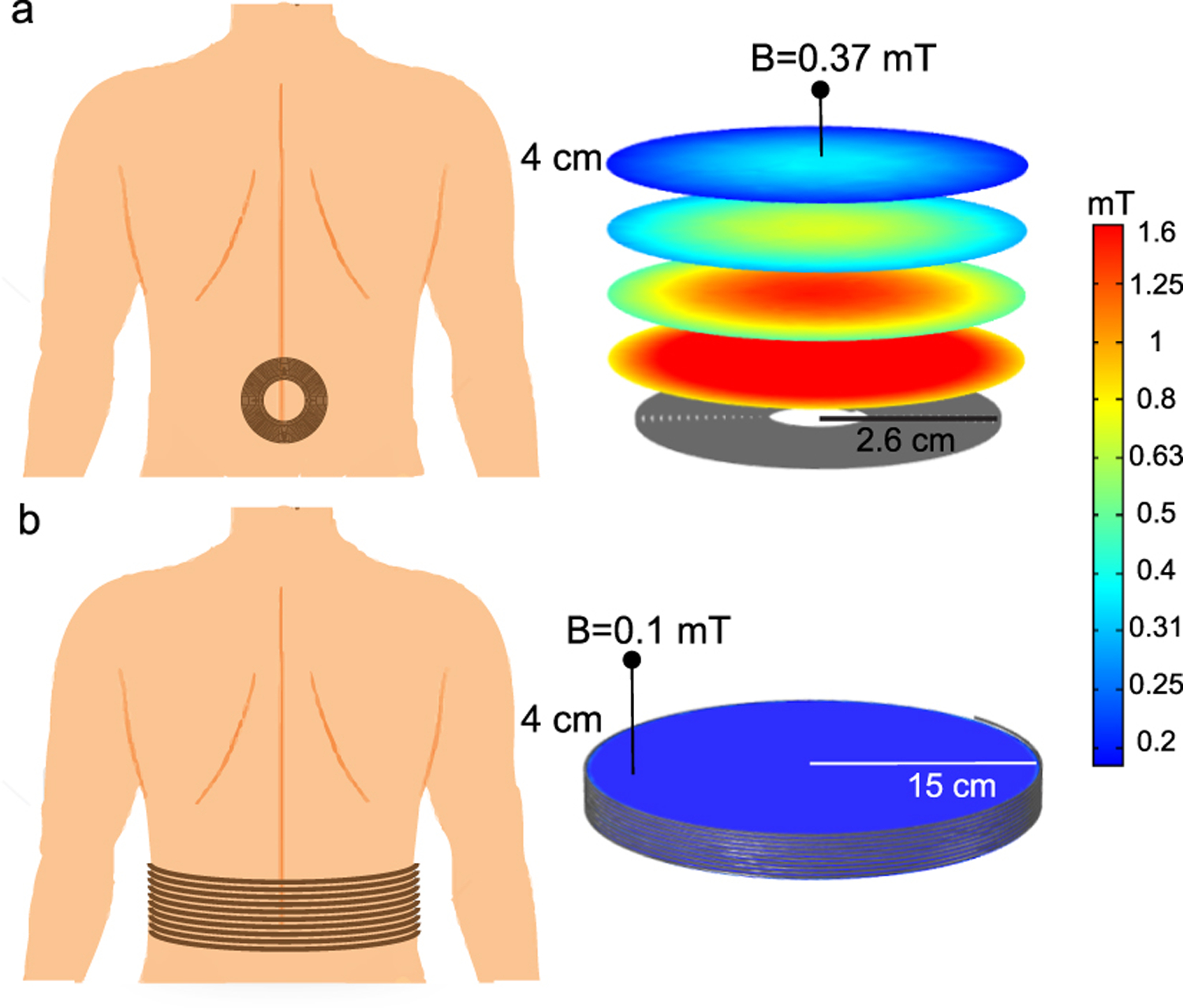
(a) Spiral coil that can be placed parallel to the implantation site and its corresponding magnetic field with 10 A excitation current. (b) Circular coil that can be wrapped around the patient waist and its corresponding magnetic field with 10 A excitation current.

To power implanted devices using a wearable system, one needs to efficiently generate a sufficient magnetic field at a distance from the coil surface, therefore, the design of the transmitter coil is specially important. For the transmitter coil, we tested using a circular coil that can be wrapped around the patient’s waist or a spiral coil that can be placed parallel to the implantation site. We built a 3D model of both coils using COMSOL multiphysics and computed the magnetic field norm values using the AC/DC module as shown in Fig. 7. The circular coil has a major radius of 15 cm whereas the spiral coil outer radius is 2.8 cm. For the same excitation current of 10 A, the spiral coil has higher magnetic field at a distance compared to the circular coil. In addition, the spiral coil has a more compact size and superior misalignment tolerance [37]–[39].

The inductance of the spiral coil can be calculated as [40]:

(4) $$L=\mu_{0}N^{2}\left(D_{out}+D_{in}\right)\left[ln\left(\frac{2.46}{\gamma }\right)+0.2\gamma^{2}\right],$$

Where N is the turns number, D_in_ is the inner diameter, D_out_ is the outer diameter, $\gamma =\frac{D_{out}-Din}{D_{out}+Din}$ and *μ*_0_ is the free space permeability= 4*π* × 10^−7^*H*/*m.T*

As mentioned in the previous sub-section, the implanted ME-BIT is sensitive to the direction of the magnetic field and reaches a maximum response when the field lines are parallel to its long axis therefore it is critical to determine the magnetic field spatial distribution and its components to place the mote at the optimal position/orientation.

**Fig. 8.**
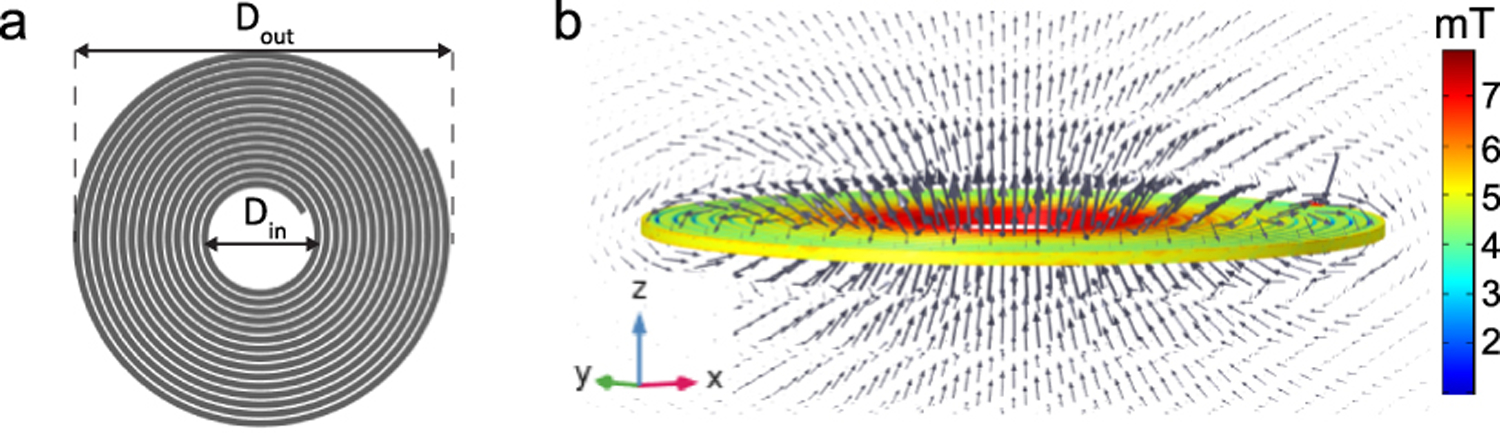
(a) 3D model of the circular spiral coil used as a transmitter coil, (b)Magnetic field distribution at the coil surface, and magnetic field lines pattern(grey arrows).

Using the model of the spiral coil shown in Fig. 7.a (15 turns, 1.5 cm inner diameter, and 5.6 cm outer diameter) we studied the spatial distribution of the generated magnetic field as can be seen in Fig. 8, the grey arrows represent the pattern of the magnetic field lines and are proportional to the field magnitude. As shown here, the magnetic field lines are parallel to the z-direction at the center of the coil and bend toward the coil edges as the radial distance from the center increases.

**Fig. 9.**
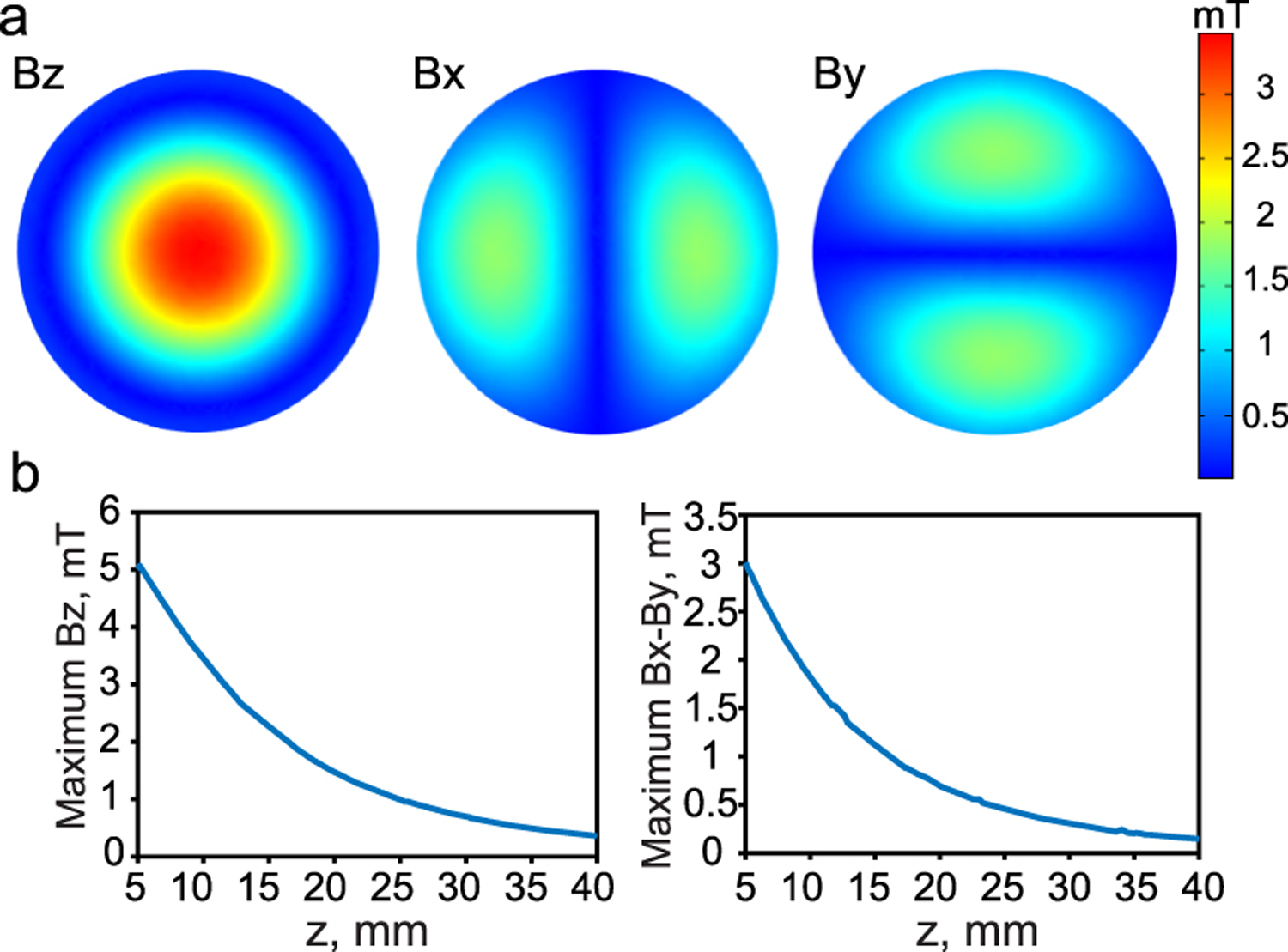
z-component, x-component, and y-component of the magnetic field generated by the transmitter coil at 1 cm vertical distance from the coil surface, (b) the maximum z-component, x-component, and y-component of the magnetic field as a function of the vertical distance from the coil surface.

We evaluated the field distribution of the magnitude of the z-, x-, and y-components at different vertical distances from the coil surface as shown in Fig. 9.

As can be seen, the z-component has a maximum value at the center of the coil and decays with an increase in the distance from the coil surface and the radial distance from the center. On the other hand, the x-component has a symmetric distribution around the y-axis and zero along the y-axis itself whereas the y-component has a symmetric distribution around the x-axis and zero along the x-axis itself. As expected, since we have a circular coil, the x and y components are of equal magnitude and the y-component distribution is shifted by 90° compared to the x-component distribution. Moreover, the x- and y-components have a lower magnitude compared to the z-component and their maximum is shifted from the center.

### III. System Performance

**Fig. 10.**
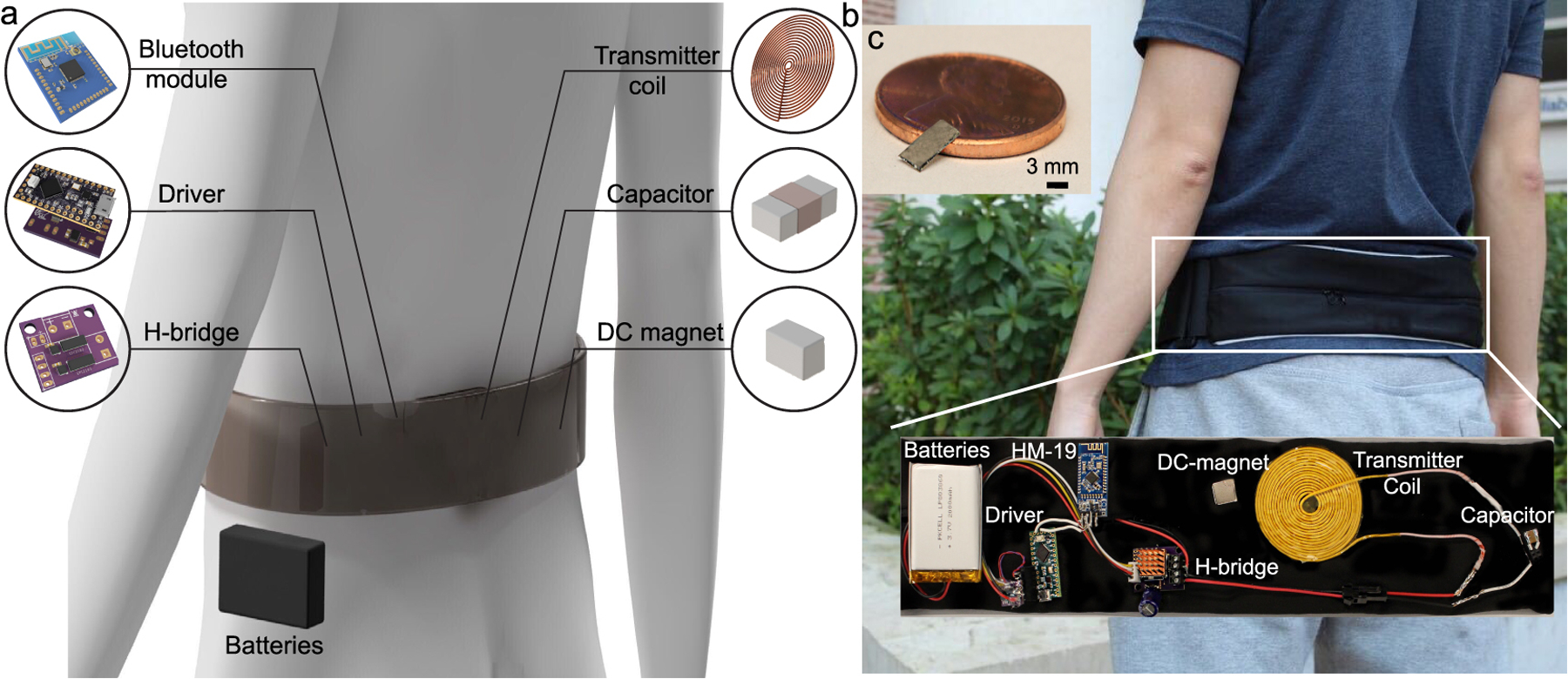
(a)System prototype, (b)physical realization of the system, (c)ME-BIT used as a receiver.

To demonstrate that we can create a wearable system capable of powering our ME-BIT implants we built a prototype transmitter as shown in Fig. 10.a. This system is powered by a set of four 3.7 V, 2000 mAh rechargeable Lithium-ion batteries as depicted in Fig. 10.a. The batteries are connected to the resonant circuit through an H-bridge that turns the input DC current into pulsed AC current based on the switching signals provided by the driver circuit. To generate the switching signals, the HM-19 module connected to the driver circuit receives the stimulation parameters set by the user through a mobile application and communicated via Bluetooth. Based on the received parameters, the Teensy LC microcontroller and custom electronics determine the timing and voltage levels to generate the switching signals accordingly. A resonant circuit of a circular spiral coil connected in series to a capacitor is used to generate the alternating magnetic field required to activate the ME film, moreover, a permanent magnet is used to provide a bias field to ensure efficient transduction.

For the proof-of-concept ME receiver, we fabricated a 7 mm*3 mm bilayer film of 30 *μ*m-thick layer of Metglas 2605SA1 (Metglas Inc) attached using HARDMAN® epoxy to a 270 *μ*m-thick layer of PZT-5A4E (Piezo Inc) as shown in Fig. 10.c.

**Fig. 11.**
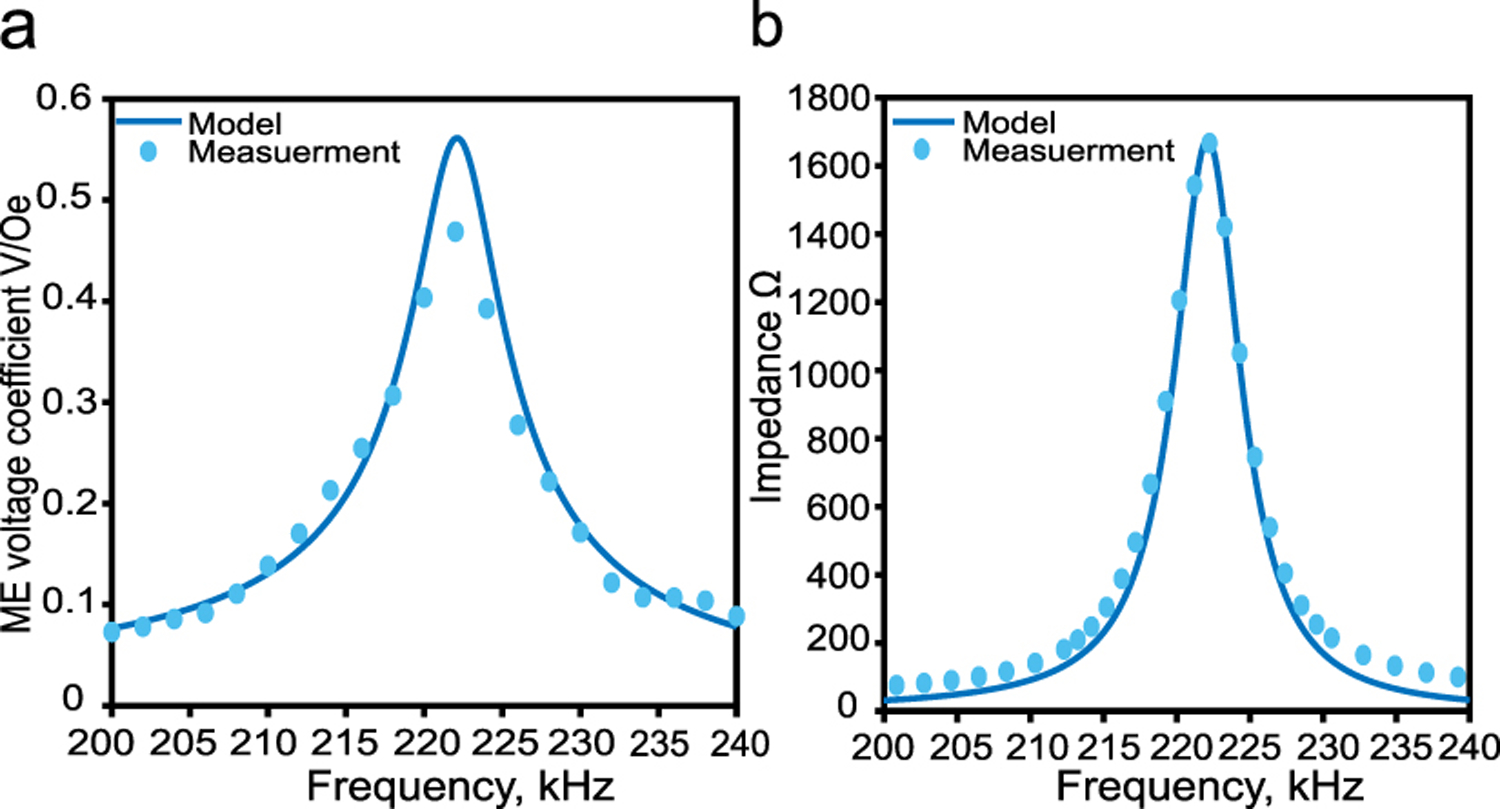
(a) Measured and calculated ME voltage coefficient of the ME film, (b) Measured and calculated real impedance of the film.

To measure the film ME voltage coefficient, we placed the film at the center of the spiral coil such that it’s long axis is parallel to the z-axis then we measured the open-circuit voltage and magnetic field strength at different frequencies to compute the ME voltage coefficient as shown in Fig. 11.a. As can be seen, the film’s mechanical resonant frequency is around 222 KHz at which it has a maximum ME voltage coefficient of 0.5 V/Oe. Fitting the measured data to the equivalent circuit model mentioned in section II.B suggests that the film has an interfacial coupling factor of 0.43 and a mechanical quality factor of 41. Fig. 11.b shows the calculated and measured real impedance of the film, at the resonance frequency, the film has a maximum resistive impedance of 1700 Ω.

To generate a magnetic field that resonates with the ME film, the pulsed current parameters are set via Bluetooth as follows: frequency = 100 Hz, pulse frequency = 222 KHz, and duty = 0.012. The pulse frequency is set based on the film resonant frequency, while the frequency and the duty values can be changed based on the application. For the resonant circuit, a coil with the same features mentioned in section II.A (*D*_*in*_=1.5 cm, *D*_*out*_=5.6 cm,N=15) is made using 1 mm thick Litz wires to minimize the skin effect. The measured inductance of the coil is 7.3 μH, hence, a 70 nF capacitor is used. This setting results in 54 mA DC current being drawn from the batteries, therefore, the system can operate continuously for up to 37 hours.

As mentioned in previous sections, the dominant component of the magnetic field at the coil’s center is the z-component, so we placed the film at the center of the coil such that its longer axis is parallel to the z-axis. In addition to the AC magnetic field, a permanent magnet is used to provide a DC bias field of 3mT. We note that for some surgical approaches it may be preferred to place the long axis of the film parallel to the skin rather than perpendicular as described here. In that case, the film can still be powered by simply shifting the coil so that the film is not directly under the coil center. In that case the fringing x or y components of the magnetic field can be used to power devices that lie parallel to the skin’s surface Fig. 9.a.

**Fig. 12.**
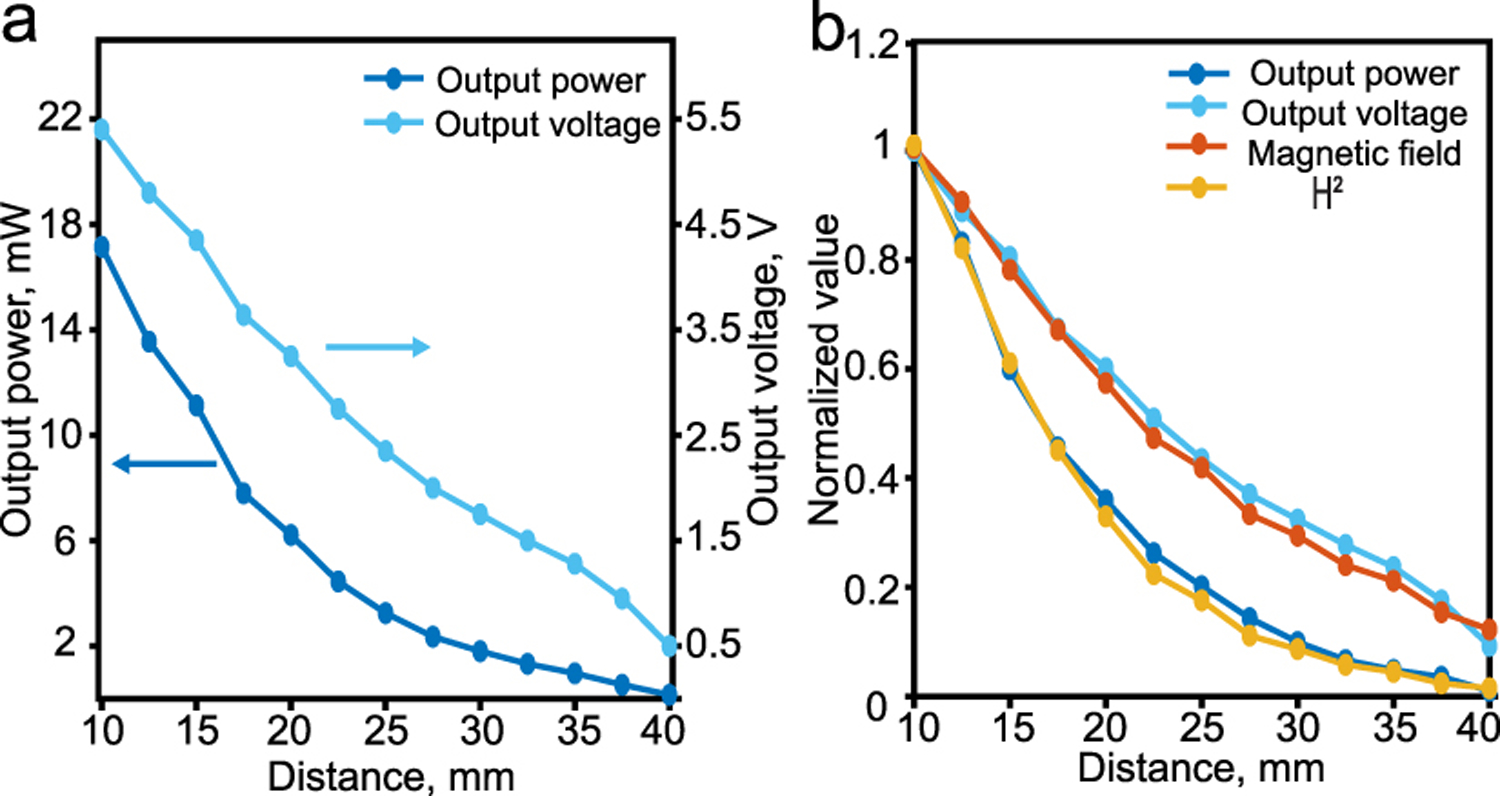
(a) Film’s output power and output voltage as a function of the distance from the coil surface, (b) Normalized values of the film output power and output voltage, and the applied magnetic field and it’s square.

To measure the maximum power, the film is connected to a resistive load of 1700 Ω, the load voltage is measured and the load power is computed as $P=\frac{V^{2}}{R}$ as shown in Fig. 12a. As the distance from the coil increases, the magnetic field decays resulting in lower output power from the ME film. Nevertheless, we can achieve an output power of 1.8 mW at a 3 cm distance where the applied magnetic field at that distance is equal to approximately 0.5 mT. Although for some applications the load impedance can not match the optimal value, an impedance matching network can be used to deliver the maximum available power.

The normalized value of the film’s output voltage, power, applied magnetic field and the square of the field as a function of the vertical distance are shown in Fig. 12.b. As mentioned in section II.B, the voltage is a linear function of the applied magnetic field whereas the power is proportional to the square of the magnetic field.

**Fig. 13.**
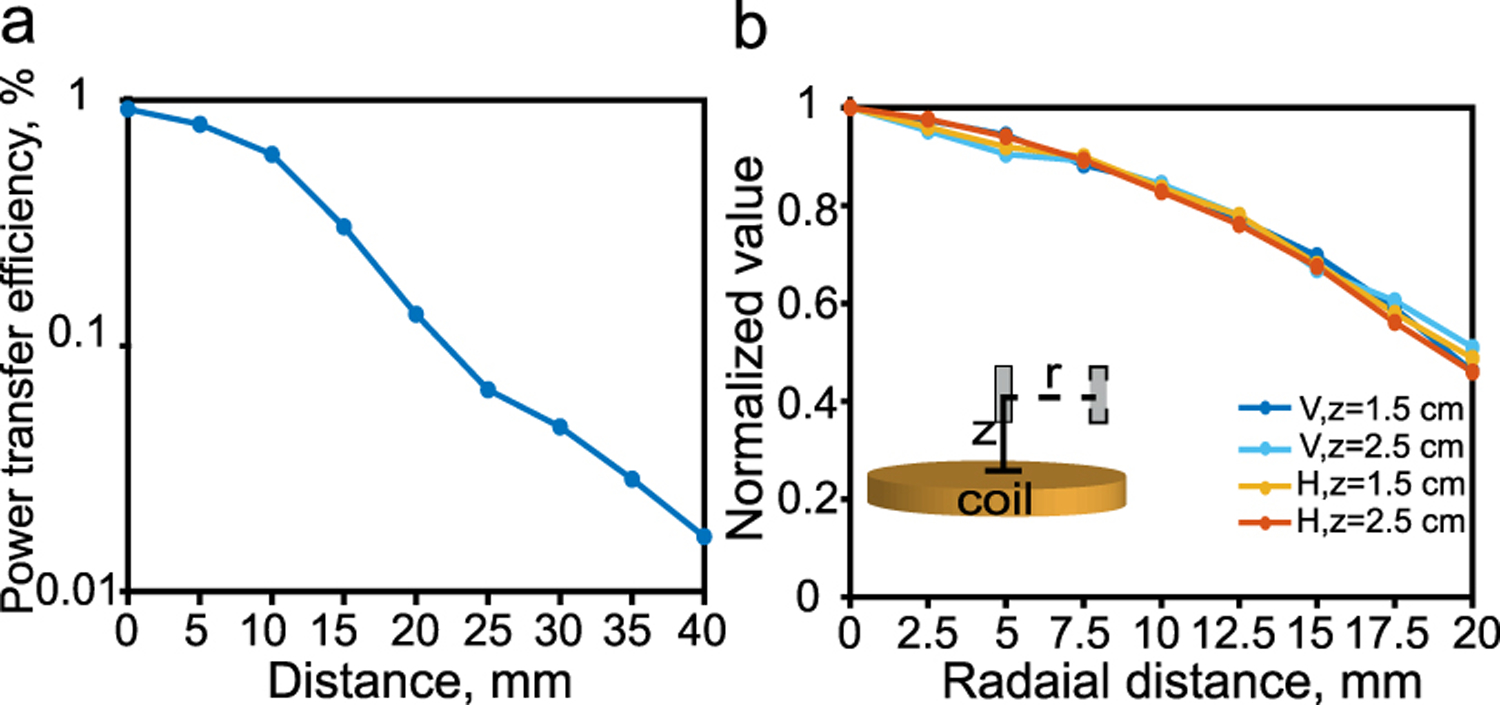
(a) Measured power transfer efficiency over various coil- film distance.(b) Normalized value of the film output voltage and applied magnetic field as a function of radial distance shift from the coil center.

The efficiency of the wireless power transfer is shown in Fig. 13.a. As can be seen, the efficiency has a maximum value of 1% at the coil surface and decays as the distance between the transmitter coil and ME film increases.

To measure how robust the power transfer efficiency is to positional misalignments we measured the film’s output voltage for different displacements relative to the optimal coupling position. Fig. 13.b shows the normalized value of the z component of the magnetic field and the output voltage of the film as it is shifted radially from the coil center at different vertical distances from the coil. The long axis of the film is maintained parallel to the z-axis, in this case, the output voltage of the film is directly related to the z component of the magnetic field which decays radially as mentioned previously. We find that 80% and 50% of the maximum voltage can still be maintained at the film terminal with 10 and 20mm shifts, respectively (Fig. 13.b). This misalignment tolerance is due to the fact that the voltage of the ME film is directly proportional to the applied ac magnetic field as mentioned in section II-A, and that the spiral coil has a high magnetic field uniformity as shown in Fig. 7.

Moreover, due to the flux concentration properties of the Metglas layer,the ME power link shows improved angular misalignment tolerance compared to inductive coupling as reported in [21].

## IV. In vivo Electrical Stimulation for Sciatic Nerve

**Fig. 14.**
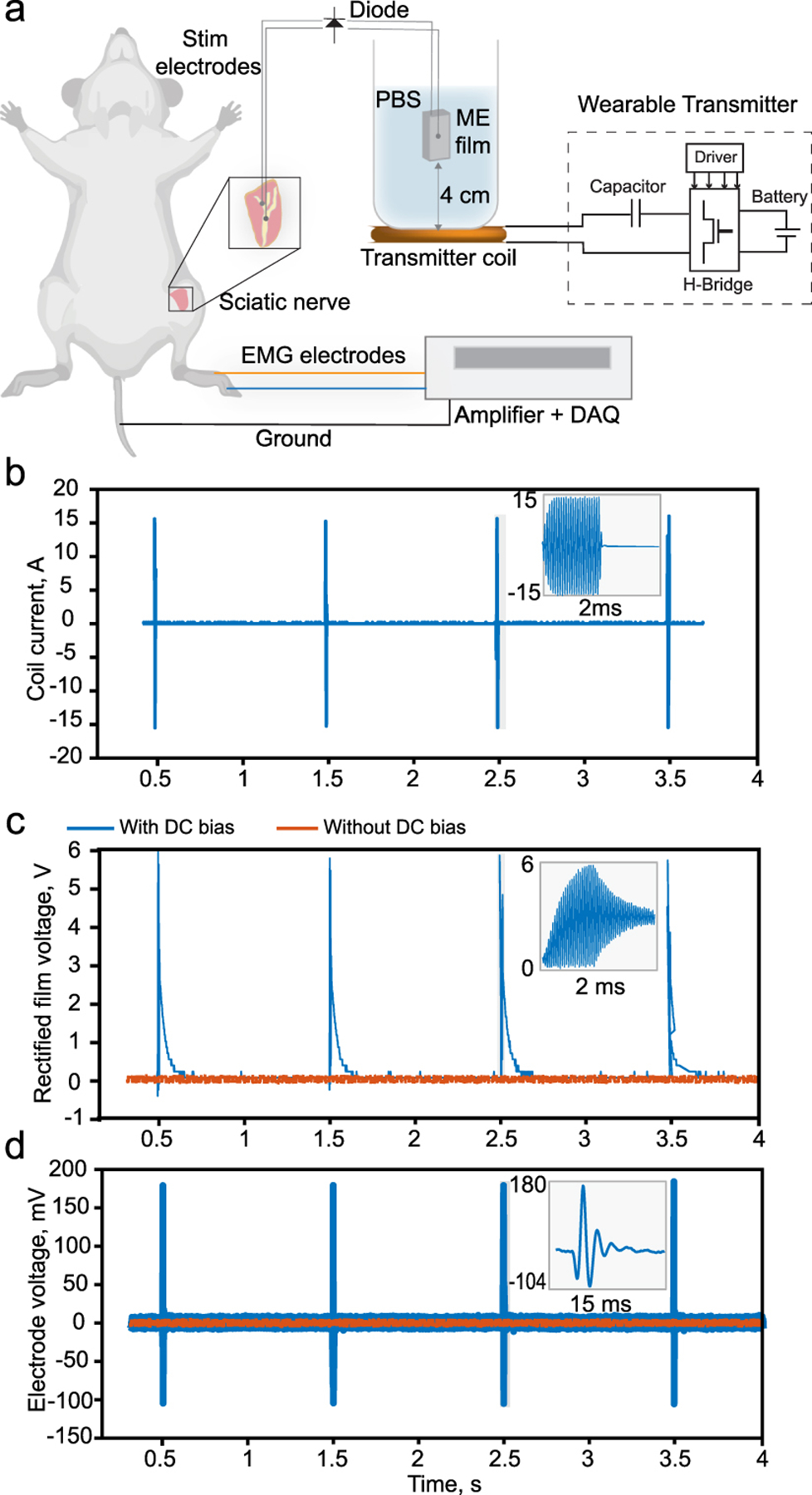
(a)Experimental setup for rat sciatic nerve stimulation and EMG recording.(b)Transmitter coil’s current.(c)Rectified ME voltage.(d)EMG elect rod’s voltage.

As a proof-of-concept, we demonstrate that our wireless transmitter can power an ME film at a distance of 4 cm in Phosphate Buffer Solution (PBS) with enough energy to stimulate rat sciatic nerve *in-vivo*. Demonstrating the ability to power an implant at a depth of 4 cm in a rodetn model is challenging due to the fact that most nerve targets are with 1 cm of the skin’s surface. Therefore, to characterize the in vivo transmitter performance at an implant depth of 4 cm, which is relevant for human applications, we used the experimental setup shown in Fig. 14.a. The ME film encapsulated with 20 *μ*m of Parylene-C is submerged in PBS at a distance of 4 cm from a transmitter coil. The film is connected to platinum electrodes that are placed into contact with the sciatic nerve of the rat. Since neurons are unresponsive to the high frequency ME voltages, we incorporated a commercial diode in parallel with the ME film to rectify the signal and modulated the field to a lower frequency (as shown previously in [19]). By pulsing the applied alternating magnetic field we were able to deliver a rectified voltage envelop of 1 Hz over the 222 kHz carrier frequency that was tuned to the film resonance (Fig. 14b–c). When we tuned the carrier frequency to the ME resonance we observed leg kick, which conformed that the voltage generated by the ME film was able to stimulate the nerve. Furthermore, we recorded the resulting compound muscle action potentials through EMG recording needles placed in the plantar muscles of the rat foot as shown in Fig. 14.d, blue line). To confirm that the stimulation was the result of the voltage produced by the ME film, we removed the DC bias magnet, which reduces the amplitude of the ME voltage. In the absence of the DC bias we observed no EMG response of leg kick (Fig. 14.d, red line).

## V. Safety Analysis

Comparing the magnetic and electric fields generated by our transmitter to the IEEE Std C95.1–2019 exposure limits we find that our transmitter is well within the range of safe exposure levels. Transmitting a time-varying magnetic field through the conductive biological tissues will induce an electric field according to Maxwell’s equations. The induced electric field drives an electrical current through the tissue resulting in heat dissipation that could be detrimental to the tissue’s health. The generated heat depends on two frequency-dependent properties of the tissue: the dielectric permittivity and the electrical conductance.

Given the potential of magnetic and electric fields to heat tissue, the IEEE Std C95.1–2019 set the limits of the electric and magnetic field exposure in the 0 Hz - 300 GHz that would ensure safe operation and protect against adverse health effects in humans [41]. The compliance with the standard implies meeting the electrostimulation limits when working below 100 kHz, the thermal limits above 5 MHz, and both limits when working in the transition region between 100 kHz and 5 MHz. The electrostimulation limit is set by the dosimetric reference limit (DRL) that is expressed as the electric field in the transition region, whereas the thermal limit is expressed by the specific absorption rate (SAR) that is defined as [41]:

(5) $$SAR=\frac{\sigma {\left|E\right|}^{2}}{\rho },$$

Where *σ* is the conductivity of the tissue (S/m), *ρ* is the density of the tissue (kg/*m*^3^), and E is the RMS electric field strength (V/m).

**Fig. 15.**
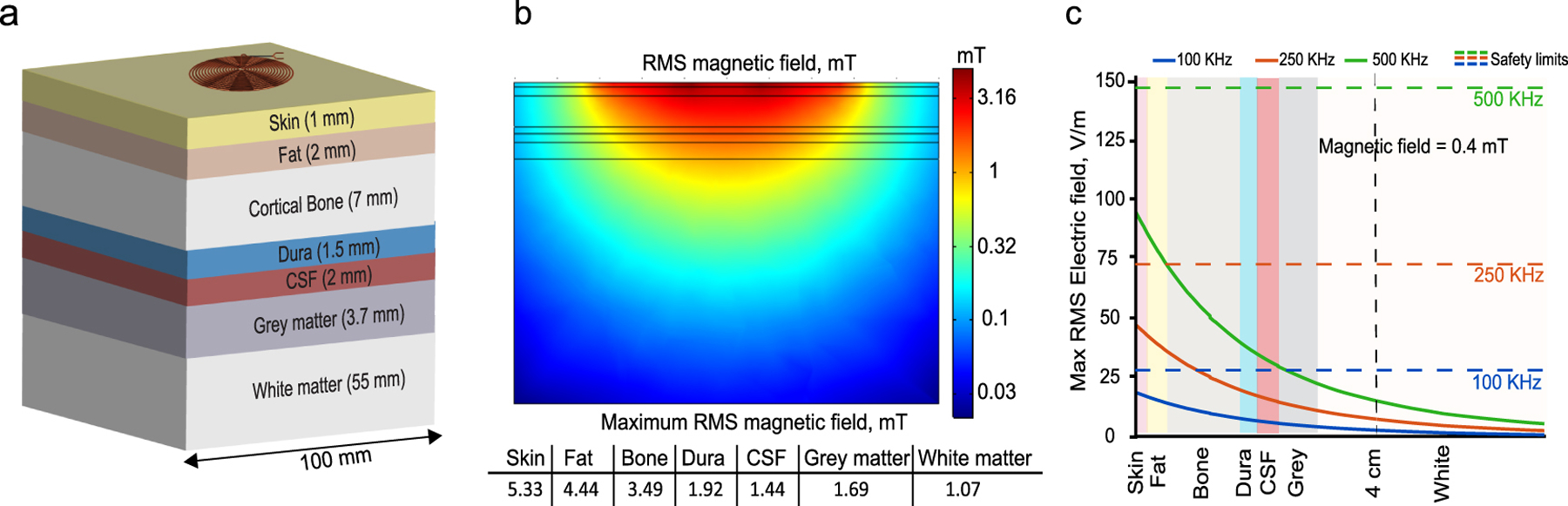
(a)Seven-layer brain tissue model,(b)RMS values of the magnetic field for a vertical cross-section at x=0 such that 0.5mT peak value is generated at 3 cm, (c)Max RMS electric field associated with magnetic field in (b) across the different brain tissues at 100, 250, 500 kHz(solid lines), IEEE Std C95.1–2019 electric field safety limits(dashed lines).

Since our system works in the 100s kHz range, both the electric field and the SAR limits should be met to ensure safe operation. To calculate these values, we modeled the seven-layers brain model reported in [42] using COMSOL. The model includes seven distinct layers of the brain tissues: skin, fat, cortical bone, dura, cerebrospinal fluid (CSF), grey matter, and white matter as shown in Fig. 15.a.

For a ME film with a length in the range of 3–10 mm, the resonance frequency is expected to be within the 100–500 kHz range as shown in Fig. 3, therefore, we analyzed the system’s compliance with the safety limits at this range. The properties of the tissues at these frequencies are listed in Table. IV (Appendix. A) [43]. As reported in the IEEE Std C95.1–2019, the limit of the SAR in unrestricted environment is 2*W*/*kg*, whereas the limit of the electric field for the head tissues for persons in unrestricted environment is given by:

(6) $$E\left(f\right)=2.94\times 10^{-4}f,$$

Where *f* is the frequency of the applied magnetic field.

Fig. 15.a shows the model of the transmitter coil and brain tissues with a transverse area of 100mm x 100mm. The magnetic fields, electric fields, and the average SAR are computed for the different layers using the magnetic and electric field modules available in COMSOL. Based on this model, we computed the magnetic field distribution through the different tissues such that a~ 0.4mT peak value is generated at 4 cm. Fig. 15.b depicts the distribution of the RMS values of the magnetic field for a vertical cross-section at x=0, at which we expect maximum field strength, moreover, the maximum values at each layer are listed.

**Fig. 16.**
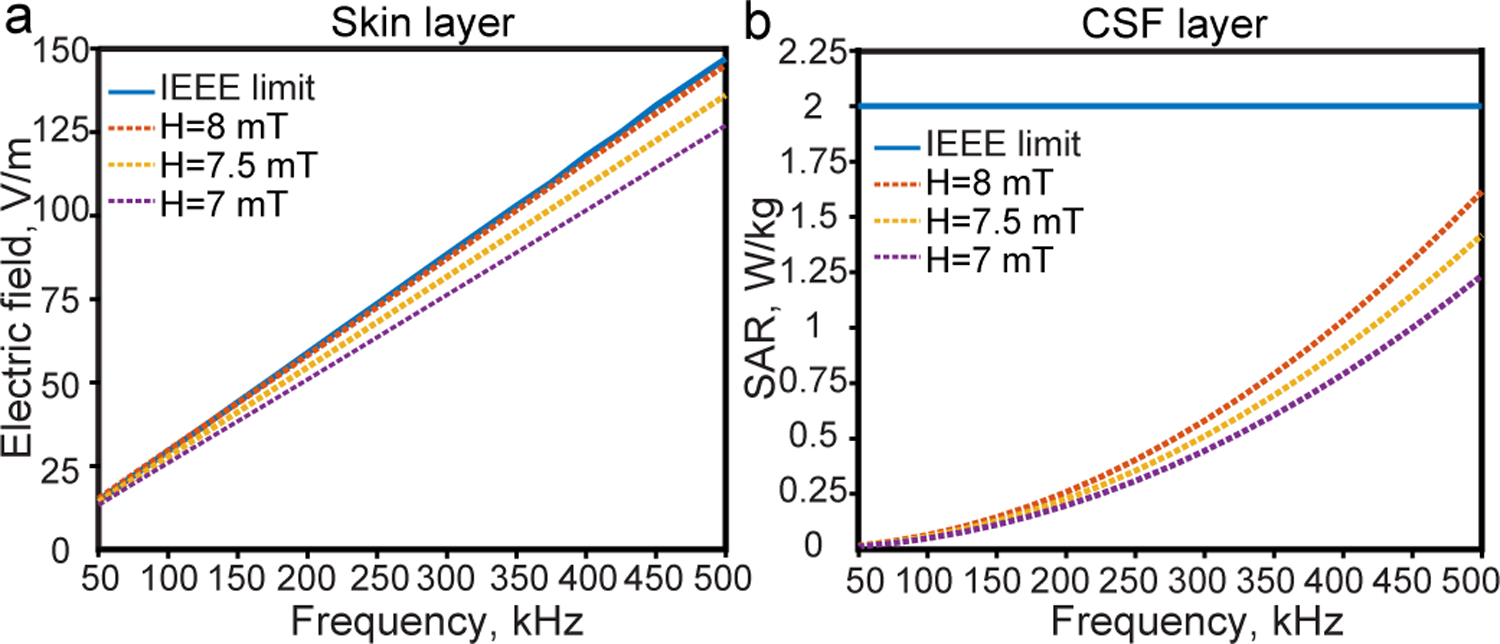
(a)Maximum electric field generated at the skin layer for different coil’s magnetic field, (b)Maximum SAR generated at the CSF layer for different coil’s magnetic field.

The generated electric field associated with this magnetic field strength depends on the operating frequency. Assuming the worst case scenario which is continuous exposure of magnetic field, the maximum value at each layer of the RMS electric field is shown in Fig. 15.c, the dashed lines represent the safety limit at each frequency, as can be seen, the electric field values are lower than the safety limits for all frequencies. The values of the SAR averaged over 10g for each layer are listed in Table. II. All values are below the safety limit of 2 W/Kg. It can be noted that the largest SAR value is for the CSF layer due to its high conductivity across the different frequencies whereas the largest electric field is generated at the skin layer as it is the closest to the coil. Further, to determine the maximum magnetic field values that can be transferred safely through the tissue in this frequency range we computed the electric field value at the skin layer and the SAR values at the CSF layer for different magnetic field exposure. As can be seen in Fig. 16, a maximum value of 8 mT can be generated at the coil surface and transmitted through tissue safely.

## VI. Conclusion

This work demonstrates a proof of principle light-weight, battery-powered wearable transmitter system that can power a miniaturized ME neural stimulator and has a battery life of 37 hr. The working principle, theory, and design of the transmitter and receiver are reported. The proposed system is capable of wirelessly powering a miniaturized 7×3 mm Metglas/PZT-5 ME receiver at a distance of 4 cm with enough engergy to stimulate the sciatic nerve of a rat *in vivo*. Moreover, the experimental results show that the system performs well under translational misalignment. We also provide some general guidelines for how the size, composition, and quality factors of ME films affect energy harvesting performance. Compared to the state-of-the-art wearable solutions for wirelessly powered implants (Table. III), our system utilizes rechargeable batteries to power the transmitter coil that generates sufficient magnetic fields to power deeply implanted miniaturized ME stimulators. Moreover, our system operates at a low-frequency range where tissue absorption of the magnetic field is negligible. Finally, using a COMSOL model of brain tissue we computed the magnetic field, electric field, and SAR values to verify the system compliance with the IEEE C95.1–2019 safety standard across the operating frequency range.

## Figures and Tables

**Table 1.** Material properties of Metglas and PZT-5 laminates.

	PZT-5	Metglas
Material density kg/m^−3^ (*ρ*_*p*_*, ρ*_*m*_)	7800	7180
Elastic compliance m^2^/N^−1^ (*s*_*p*_*, s*_*m*_)	19.2 × 10^−12^	9.09 × 10^−12^
Piezoelectric/piezomagnetic coefficient m/V^−1^,Wb/N^−1^ (*d*_*p,*31_*, d*_*m,*33_)	−190 × 10^−12^	8.25 × 10^−9^
Relative permittivity/permeability (*ε*_*p*_*, μ*_*m*_)	1800	45000

**Table 2.** Computed average SAR (W/Kg^−1^) values over 10 g at 100, 250, 500 for each layer.

	100k	250k	500k
Skin	3.4 × 10^−5^	6.8 × 10^−4^	8.1 × 10^−3^
Fat	3.0 × 10^−3^	1.9 × 10^−2^	7.5×10^−2^
Bone	5.0 × 10^−5^	3.2 × 10^−4^	1.3 × 10^−2^
Dura	7.9 × 10^−3^	4.9 × 10^−2^	19.4 × 10^−2^
CSF	2.6 × 10^−2^	17.2 × 10^−2^	68.8 × 10^−2^
Grey matter	7.2 × 10^−4^	4.8 × 10^−3^	2.0 × 10^−2^
White matter	2.5 × 10^−6^	1.7 × 10^−5^	7.2 × 10^−5^

**Table 3.** Comparison with state of the art wearable systems for wireless powered implants.

	This work	[44]	[45]	[46]	[47]
Application	Neural stimulation	Gastro stimulation	Cardiac pacing	Capsule endoscopy	Neural recording
Wireless link	Magnetoelectric	Inductive coupling	RF	Inductive coupling	Ultrasonic
Operating frequency	240 kHz	1.3 MHz	954 MHz	250 kHz	1.8 MHz
Implant size	21 mm^2^	350 mm^2^	120 mm^2^	113 mm^2^	0.56 mm^2^
Operating distance	4 cm	4 cm	11 cm	1 cm	1.4 cm
Power source	Battery	Battery	Signal generator and power amplifier	DC supply	Battery
WPT efficiency	1 %	3.5 %	8 %	8 %	N/A
Test model	Rat	Saline	Ovine	Air	Tissue phantom
Battery Lifetime	37 h	N/A	N/A	N/A	1.25 h

**Table 4.** Brain tissues properties at 100, 250, 500 khz for each layer.

		Frequency = 100kHz		Frequency = 250kHz		Frequency = 500kHz	
Tissue	Density kg/m^−3^	*ε*_*r*_	*σ*	*ε*_*r*_	*σ*	*ε*_*r*_	*σ*
Skin	1109	1.12 × 10^3^	4.5 × 10^−4^	1.1 × 10^3^	1.46 × 10^−3^	1.06 × 10^3^	4.36 × 10^−3^
Fat	911	1.01 × 10^2^	4.34 × 10^−2^	6.78 × 10^1^	4.36 × 10^−2^	5.68 × 10^1^	4.38 × 10^−2^
Cortical bone	1908	2.28 × 10^2^	2.08 × 10^−2^	1.97 × 10^2^	2.12 × 10^−2^	1.75 × 10^2^	2.22 × 10^−2^
Dura	1174	3.26 × 10^2^	5.02 × 10^−1^	2.82 × 10^2^	5.02 × 10^−1^	2.64 × 10^2^	5.03 × 10^−1^
CSF	1007	1.09 × 10^2^	2	1.09 × 10^2^	2	1.09 × 10^2^	2
Grey matter	1045	3.22 × 10^3^	1.34 × 10^−1^	1.75 × 10^3^	1.43 × 10^−1^	1.19 × 10^3^	1.52 × 10^−1^
White matter	1041	2.11 × 10^3^	8.18 × 10^−2^	1.11 × 10^3^	8.85 × 10^−2^	7.12 × 10^2^	9.47 × 10^−2^

## Appendix A Brain tissues properties.

The density, relative permittivity, and conductivity of the brain tissues are listed in Table. IV.

## References

[1] Vitale F and Litt B, “Bioelectronics: the promise of leveraging the body’s circuitry to treat disease,” Bioelectronics in Medicine, vol. 1, pp. 3–7, Dec. 2017. Publisher: Future Medicine.

[2] “Durable miniaturized bioelectronics,” Nature Biomedical Engineering, vol. 1, pp. 1–1, Mar. 2017. Number: 3 Publisher: Nature Publishing Group.

[3] “Fitter in-body bioelectronics,” Nature Biomedical Engineering, vol. 3, pp. 1–2, Jan. 2019. Number: 1 Publisher: Nature Publishing Group. 10.1038/s41551-018-0346-3 30932075

[4] Lorach H, Goetz G, Smith R, Lei X, Mandel Y, Kamins T, Mathieson K, Huie P, Harris J, Sher A, and Palanker D, “Photovoltaic restoration of sight with high visual acuity,” Nature Medicine, vol. 21, pp. 476–482, May 2015. Number: 5 Publisher: Nature Publishing Group. 10.1038/nm.3851 PMC4601644 25915832

[5] Cianchetti M, Laschi C, Menciassi A, and Dario P, “Biomedical applications of soft robotics,” Nature Reviews Materials, vol. 3, pp. 143–153, June 2018. Number: 6 Publisher: Nature Publishing Group.

[6] Afanasenkau D, Kalinina D, Lyakhovetskii V, Tondera C, Gorsky O, Moosavi S, Pavlova N, Merkulyeva N, Kalueff AV, Minev IR, and Musienko P, “Rapid prototyping of soft bioelectronic implants for use as neuromuscular interfaces,” Nature Biomedical Engineering, vol. 4, pp. 1010–1022, Oct. 2020. Number: 10 Publisher: Nature Publishing Group. 10.1038/s41551-020-00615-7 32958898

[7] Bhave G, Chen JC, Singer A, Sharma A, and Robinson JT, “Distributed sensor and actuator networks for closed-loop bioelectronic medicine,” Materials Today, vol. 46, pp. 125–135, June 2021. 34366697 10.1016/j.mattod.2020.12.020 PMC8336425

[8] Fan B and Li W, “Miniaturized optogenetic neural implants: a review,” Lab on a Chip, vol. 15, no. 19, pp. 3838–3855, 2015. Publisher: Royal Society of Chemistry. 26308721 10.1039/c5lc00588d

[9] Das R, Moradi F, and Heidari H, “Biointegrated and Wirelessly Powered Implantable Brain Devices: A Review,” IEEE transactions on biomedical circuits and systems, vol. 14, pp. 343–358, Apr. 2020. 31944987 10.1109/TBCAS.2020.2966920

[10] Deer TR, Naidu R, Strand N, Sparks D, Abd-Elsayed A, Kalia H, Hah JM, Mehta P, Sayed D, and Gulati A, “A review of the bioelectronic implications of stimulation of the peripheral nervous system for chronic pain conditions,” Bioelectronic Medicine, vol. 6, p. 9, Apr. 2020. 32346553 10.1186/s42234-020-00045-5 PMC7181529

[11] Williams NR and Okun MS, “Deep brain stimulation (DBS) at the interface of neurology and psychiatry,” Nov. 2013. Publisher: American Society for Clinical Investigation. 10.1172/JCI68341 PMC3809784 24177464

[12] Cocchi L and Zalesky A, “Personalized Transcranial Magnetic Stimulation in Psychiatry,” Biological Psychiatry: Cognitive Neuroscience and Neuroimaging, vol. 3, pp. 731–741, Sept. 2018. 29571586 10.1016/j.bpsc.2018.01.008

[13] Deer TR and Stewart CD, “Complications of Spinal Cord Stimulation: Identification, Treatment, and Prevention,” Pain Medicine, vol. 9, pp. S93–S101, May 2008.

[14] Piech DK, Johnson BC, Shen K, Ghanbari MM, Li KY, Neely RM, Kay JE, Carmena JM, Maharbiz MM, and Muller R, “A wireless millimetre-scale implantable neural stimulator with ultrasonically powered bidirectional communication,” Nature Biomedical Engineering, vol. 4, pp. 207–222, Feb. 2020. Number: 2 Publisher: Nature Publishing Group. 10.1038/s41551-020-0518-9 32076132

[15] Mujeeb-U-Rahman M, Adalian D, Chang C-F, and Scherer A, “Optical power transfer and communication methods for wireless implantable sensing platforms,” Journal of Biomedical Optics, vol. 20, p. 095012, Sept. 2015. 26405820 10.1117/1.JBO.20.9.095012

[16] Liu C, Guo Y-X, Sun H, and Xiao S, “Design and Safety Considerations of an Implantable Rectenna for Far-Field Wireless Power Transfer,” IEEE Transactions on Antennas and Propagation, vol. 62, pp. 5798–5806, Nov. 2014. Conference Name: IEEE Transactions on Antennas and Propagation.

[17] Munshi R, Qadri SM, Zhang Q, Castellanos Rubio I, del Pino P, and Pralle A, “Magnetothermal genetic deep brain stimulation of motor behaviors in awake, freely moving mice,” eLife, vol. 6, p. e27069, Aug. 2017. Publisher: eLife Sciences Publications, Ltd. 28826470 10.7554/eLife.27069 PMC5779110

[18] Chen R, Romero G, Christiansen MG, Mohr A, and Anikeeva P, “Wireless magnetothermal deep brain stimulation,” Science, vol. 347, pp. 1477–1480, Mar. 2015. Publisher: American Association for the Advancement of Science Section: Report. 25765068 10.1126/science.1261821

[19] Singer A, Dutta S, Lewis E, Chen Z, Chen JC, Verma N, Avants B, Feldman AK, O’Malley J, Beierlein M, Kemere C, and Robinson JT, “Magnetoelectric materials for miniature, wireless neural stimulation at therapeutic frequencies,” bioRxiv, p. 461855, Mar. 2020. Publisher: Cold Spring Harbor Laboratory Section: New Results. 10.1016/j.neuron.2020.05.019 PMC7818389 32516574

[20] Yu Z, Chen JC, Avants BW, He Y, Singer A, Robinson JT, and Yang K, “34.3 An 8.2mm3 Implantable Neurostimulator with Magnetoelectric Power and Data Transfer,” in 2020 IEEE International Solid-State Circuits Conference - (ISSCC), pp. 510–512, Feb. 2020. ISSN: 2376-8606.

[21] Yu Z, Chen JC, Alrashdan FT, Avants BW, He Y, Singer A, Robinson JT, and Yang K, “MagNI: A Magnetoelectrically Powered and Controlled Wireless Neurostimulating Implant,” IEEE Transactions on Biomedical Circuits and Systems, pp. 1–1, 2020. Conference Name: IEEE Transactions on Biomedical Circuits and Systems. 10.1109/TBCAS.2020.3037862 PMC8712272 33180732

[22] Freeman DK, O’Brien JM, Kumar P, Daniels B, Irion RA, Shraytah L, Ingersoll BK, Magyar AP, Czarnecki A, Wheeler J, Coppeta JR, Abban MP, Gatzke R, Fried SI, Lee SW, Duwel AE, Bernstein JJ, Widge AS, Hernandez-Reynoso A, Kanneganti A, Romero-Ortega MI, and Cogan SF, “A Sub-millimeter, Inductively Powered Neural Stimulator,” Frontiers in Neuroscience, vol. 11, 2017. Publisher: Frontiers. 10.3389/fnins.2017.00659 PMC5712043 29230164

[23] Singer A and Robinson JT, “Wireless Power Delivery Techniques for Miniature Implantable Bioelectronics,” Advanced Healthcare Materials, vol. n/a, no. n/a, p. 2100664. _eprint: 10.1002/adhm.202100664. PMC8754427 34114368

[24] Thimot J and Shepard KL, “Bioelectronic devices: Wirelessly powered implants,” Nature Biomedical Engineering, vol. 1, pp. 1–2, Mar. 2017. Number: 3 Publisher: Nature Publishing Group.

[25] Yu Z, Chen JC, He Y, Alrashdan FT, Avants BW, Singer A, Robinson JT, and Yang K, “Multisite bio-stimulating implants magnetoelectrically powered and individually programmed by a single transmitter,” in 2021 IEEE Custom Integrated Circuits Conference (CICC), pp. 1–2, Apr. 2021. ISSN: 2152-3630.

[26] Nan T, Lin H, Gao Y, Matyushov A, Yu G, Chen H, Sun N, Wei S, Wang Z, Li M, Wang X, Belkessam A, Guo R, Chen B, Zhou J, Qian Z, Hui Y, Rinaldi M, McConney ME, Howe BM, Hu Z, Jones JG, Brown GJ, and Sun NX, “Acoustically actuated ultra-compact NEMS magnetoelectric antennas,” Nature Communications, vol. 8, p. 296, Aug. 2017. Number: 1 Publisher: Nature Publishing Group. 10.1038/s41467-017-00343-8 PMC5567369 28831042

[27] Zaeimbashi M, Nasrollahpour M, Khalifa A, Romano A, Liang X, Chen H, Sun N, Matyushov A, Lin H, Dong C, Xu Z, Mittal A, Martos-Repath I, Jha G, Mirchandani N, Das D, Onabajo M, Shrivastava A, Cash S, and Sun NX, “Ultra-compact Dual-band Smart NEMS Magnetoelectric Antennas for Simultaneous Wireless Energy Harvesting and Magnetic Field Sensing,” bioRxiv, p. 2020.06.22.165894, June 2020. Publisher: Cold Spring Harbor Laboratory Section: New Results. 10.1038/s41467-021-23256-z PMC8149822 34035237

[28] Cata JP, Cordella JV, Burton AW, Hassenbusch SJ, Weng H-R, and Dougherty PM, “Spinal cord stimulation relieves chemotherapy-induced pain: a clinical case report,” Journal of Pain and Symptom Management, vol. 27, pp. 72–78, Jan. 2004. 14711471 10.1016/j.jpainsymman.2003.05.007

[29] Kaniusas E, Kampusch S, Tittgemeyer M, Panetsos F, Gines RF, Papa M, Kiss A, Podesser B, Cassara AM, Tanghe E, Samoudi AM, Tarnaud T, Joseph W, Marozas V, Lukosevicius A, Ištuk N, Lechner S, Klonowski W, Varoneckas G, Széles JC, and Šarolić A, “Current Directions in the Auricular Vagus Nerve Stimulation II – An Engineering Perspective,” Frontiers in Neuroscience, vol. 13, p. 772, July 2019. 31396044 10.3389/fnins.2019.00772 PMC6667675

[30] Kampusch S, Kaniusas E, and Széles JC, “New approaches in multipunctual percutaneous stimulation of the auricular vagus nerve,” in 2013 6th International IEEE/EMBS Conference on Neural Engineering (NER), pp. 263–266, Nov. 2013. ISSN: 1948-3554.

[31] Dapino MJ, “On magnetostrictive materials and their use in adaptive structures,” Structural Engineering and Mechanics, vol. 17, pp. 303–329, Mar. 2004.

[32] Tu C, Chu Z-Q, Spetzler B, Hayes P, Dong C-Z, Liang X-F, Chen H-H, He Y-F, Wei Y-Y, Lisenkov I, Lin H, Lin Y-H, McCord J, Faupel F, Quandt E, and Sun N-X, “Mechanical-Resonance-Enhanced Thin-Film Magnetoelectric Heterostructures for Magnetometers, Mechanical Antennas, Tunable RF Inductors, and Filters,” Materials, vol. 12, July 2019. 10.3390/ma12142259 PMC6679207 31337062

[33] Dong S, Li J-F, and Viehland D, “Magnetoelectric coupling, efficiency, and voltage gain effect in piezoelectric-piezomagnetic laminate composites,” Journal of Materials Science, vol. 41, pp. 97–106, Jan. 2006.

[34] Truong BD, “Fundamental Issues in Magnetoelectric Transducers: Magnetic Field Sensing Versus Wireless Power Transfer Systems,” IEEE Sensors Journal, vol. 20, pp. 5322–5328, May 2020. Conference Name: IEEE Sensors Journal.

[35] Filippov DA, Galichyan TA, and Laletin VM, “Influence of an interlayer bonding on the magnetoelectric effect in the layered magnetostrictive-piezoelectric structure,” Applied Physics A, vol. 116, pp. 2167–2171, Sept. 2014.

[36] Yu H, Zeng M, Wang Y, Wan JG, and Liu J-M, “Magnetoelectric resonance-bandwidth broadening of Terfenol-D/epoxy-Pb(Zr,Ti)O3 bilayers in parallel and series connections,” Applied Physics Letters, vol. 86, p. 032508, Jan. 2005. Publisher: American Institute of Physics.

[37] Imura T, “Magnetic Resonance Coupling Phenomenon and Basic Characteristics,” in Wireless Power Transfer: Using Magnetic and Electric Resonance Coupling Techniques (Imura T, ed.), pp. 71–92, Singapore: Springer, 2020.

[38] Orekan T, Zhang P, and Shih C, “Analysis, Design, and Maximum Power-Efficiency Tracking for Undersea Wireless Power Transfer,” IEEE Journal of Emerging and Selected Topics in Power Electronics, vol. 6, pp. 843–854, June 2018.

[39] Sampath JPK, Alphones A, and Shimasaki H, “Coil design guidelines for high efficiency of wireless power transfer (WPT),” in 2016 IEEE Region 10 Conference (TENCON), pp. 726–729, Nov. 2016. ISSN: 2159-3450.

[40] Nguyen MQ, Hughes Z, Woods P, Seo Y-S, Rao S, and Chiao J-C, “Field Distribution Models of Spiral Coil for Misalignment Analysis in Wireless Power Transfer Systems,” IEEE Transactions on Microwave Theory and Techniques, vol. 62, pp. 920–930, Apr. 2014. Conference Name: IEEE Transactions on Microwave Theory and Techniques.

[41] “IEEE Standard for Safety Levels with Respect to Human Exposure to Electric, Magnetic, and Electromagnetic Fields, 0 Hz to 300 GHz,” IEEE Std C95.1–2019 (Revision of IEEE Std C95.1–2005/ Incorporates IEEE Std C95.1–2019/Cor 1–2019), pp. 1–312, Oct. 2019. Conference Name: IEEE Std C95.1–2019 (Revision of IEEE Std C95.1–2005/ Incorporates IEEE Std C95.1–2019/Cor 1–2019).

[42] Hout S and Chung J-Y, “Design and Characterization of a Miniaturized Implantable Antenna in a Seven-Layer Brain Phantom,” IEEE Access, vol. 7, pp. 162062–162069, 2019. Conference Name: IEEE Access.

[43] Hasgall P, Di Gennaro F, Baumgartner C, Neufeld E, Gosselin M, Payne D, Klingenböck A, and Kuster N, “It’is database for thermal and electromagnetic parameters of biological tissues,” Version 3.0, 2015.

[44] Seo Y-S, Hughes Z, Isom D, Nguyen MQ, Deb S, Rao S, and Chiao J-C, “Wireless power transfer for a miniature gastrostimulator,” in 2012 42nd European Microwave Conference, pp. 229–232, Oct. 2012.

[45] Asif SM, Iftikhar A, Hansen JW, Khan MS, Ewert DL, and Braaten BD, “A Novel RF-Powered Wireless Pacing via a Rectenna-Based Pacemaker and a Wearable Transmit-Antenna Array,” IEEE Access, vol. 7, pp. 1139–1148, 2019. Conference Name: IEEE Access.

[46] Basar MR, Ahmad MY, Cho J, and Ibrahim F, “An Improved Wearable Resonant Wireless Power Transfer System for Biomedical Capsule Endoscope,” IEEE Transactions on Industrial Electronics, vol. 65, pp. 7772–7781, Oct. 2018. Conference Name: IEEE Transactions on Industrial Electronics.

[47] Piech DK, Kay JE, Boser BE, and Maharbiz MM, “Rodent wearable ultrasound system for wireless neural recording,” in 2017 39th Annual International Conference of the IEEE Engineering in Medicine and Biology Society (EMBC), pp. 221–225, July 2017. ISSN: 1558-4615. 10.1109/EMBC.2017.8036802 29059850

